# Evaluation of Connectivity Reliability for Heterogeneous Functional Chain Networks Considering Dynamic Reconfiguration

**DOI:** 10.3390/s26123893

**Published:** 2026-06-18

**Authors:** Yunlong Bian, Junhai Cao, Chengming He, Haidong Du, Zhenwei Wang, Xiaofeng Yue

**Affiliations:** 1 Army Arms University of PLA, Beijing 100072, Chinachengminghe@163.com (C.H.); hdongd@163.com (H.D.); zwwang@163.com (Z.W.); xiaofy@163.com (X.Y.); 2 National Key Laboratory of Intelligent Parallel Technology, Beijing 100072, China

**Keywords:** heterogeneous network, dynamic reconfiguration, connectivity reliability, simulation

## Abstract

The increasing diversity and complexity of modern mission scenarios have led to growing heterogeneity among nodes in mobile ad hoc networks: node functions, onboard devices, and operational parameters are becoming more diverse, and inter-node links are correspondingly no longer homogeneous. Such networks, termed heterogeneous functional chain networks, orchestrate nodes with distinct functions into multiple functional chains that cooperate to accomplish the overall mission. Accordingly, the evaluation of connectivity reliability in these networks has shifted from a topology-oriented paradigm to a functional structure-oriented one. This paper investigates the impact of dynamic reconfiguration mechanisms on the connectivity reliability of heterogeneous functional chain networks, accounting for node failures, node mobility, and link reliability. A Dynamic Reconfiguration Scheme (DRS) is designed based on the principles of minimum movement and minimum-ordinal decision node, and a suite of evaluation metrics—including normalized connectivity reliability, network quality, and connectivity reliability—is proposed together with a Monte Carlo simulation algorithm. The proposed approach is validated via MATLAB simulations involving 210 heterogeneous nodes organized into 70 functional chains. Results demonstrate that dynamic reconfiguration increases the terminal number of functional chains by 170.83% (from 12.10 ± 0.673 to 32.77 ± 2.241), improves normalized connectivity reliability by 170.73% (from 0.1729 ± 0.010 to 0.4681 ± 0.032), and enhances network quality by 82.96%. The connectivity reliability is further shown to evolve through three distinct temporal stages: an initial stable period where functional chains remain largely intact, a mid-stage fluctuation period characterized by iterative destruction–reconfiguration dynamics, and a late-stage degradation period triggered by candidate node pool depletion.

## 1. Introduction

Mobile ad hoc networks (MANETs) exist in various forms such as wireless sensor networks, unmanned aerial vehicle (UAV) swarms, vehicular ad hoc networks, and more. They are utilized in diverse mission scenarios, including low-altitude logistics, post-disaster emergency communication, precision agriculture, forest fire monitoring, electric power line inspection, search and rescue, and UAV cooperative reconnaissance [1,2,3]. These application scenarios often lack network infrastructure or are far from urban areas, and they impose higher requirements on the functionality and reliability of the network for executing specific tasks. For instance, the task network needs to possess capabilities such as temperature and humidity sensing; fire source identification; and emergency suppression, video transmission and analysis, and it must ensure communication connectivity between nodes at all times within a specified time frame to complete information exchange. Evidently, while MANETs achieve significant social and economic benefits, they rely on the reliable operation of various types of nodes within the network and the timely and accurate transmission of information between nodes. In the study of complex networks, connectivity reliability [4] is directly related to the success of network tasks: from the perspective of network deployment and application, only when the network maintains connectivity throughout the entire task cycle and at all stages, and its performance is above the task baseline, can the network fully exert its functions and achieve social and economic value, such as timely maintenance of various wireless sensor networks and emergency communication networks for detection to cope with emergencies; from the perspective of network attack, only by timely disrupting key network nodes or connectivity links between nodes, causing the network topology to collapse, can the overall impact of the network be minimized, such as disrupting epidemic transmission networks and rumor spread networks. Thus, the study of connectivity reliability has a dual nature of “shield and spear” and reflects a dynamic balance: accurately assessing the connectivity reliability of critical functional networks, deploying and assigning tasks to the network according to actual conditions, and proposing targeted improvement measures for weak links and key influencing factors in the network is the “shield” and “defense”; precisely analyzing important nodes and links in negative functional networks to achieve maximum damage to harmful networks with minimal attack costs is the “spear” and “attack”.

From the perspective of system reliability theory, reliability is defined as the ability of a product to perform specified functions under specified conditions for a specified time [5]. For an individual product, reliability is considered an inherent attribute established during the design and manufacturing stages. However, when multiple heterogeneous products are assembled into a network system, a critical distinction emerges: network systems are often purpose-built and deployed in response to specific mission requirements. Different mission profiles entail different operating conditions, mission durations, and required network functionalities. Consequently, network reliability must be evaluated on a per-mission or per-mission-class basis rather than treated as a fixed, invariant system property. This distinction is particularly pronounced for MANETs, which exhibit several distinctive attributes: the absence of fixed infrastructure, self-configuration capability, dynamic topology variation, device-to-device or ad hoc routing-based communication, inherent topological instability, susceptibility to failure propagation, and constraints on energy and bandwidth. These attributes cause MANET reliability to exhibit temporal fluctuations. More importantly, even in the absence of external interventions such as maintenance or node replenishment, a MANET possesses intrinsic self-recovery and self-adaptation capabilities: mechanisms such as dynamic reconfiguration can reshape network topology and inter-node connectivity, thereby affecting network performance and functional capacity. In contrast to classical system reliability—which is primarily determined during the design and manufacturing stages and treated as an inherent system attribute—the reliability of a MANET is not a static, once-established property. Rather, it is dynamically shaped throughout the system lifecycle by runtime management mechanisms, including dynamic reconfiguration, resource scheduling, and adaptive topology control. Network reliability, in this sense, is not given—it is sustained.

In traditional homogeneous control networks [4], a few critical nodes are responsible for decision-making and control. The failure of a single decision node can disrupt information transmission for an entire cluster or even cause the entire network to collapse. In contrast, the decision-making and control functions in heterogeneous networks are distributed across specific types of nodes, which work in concert to form functional chains. These chains are coupled together to constitute a functional network [4]. When any node within a functional chain fails, the entire chain is considered faulty, as it can no longer deliver its complete function. However, the self-organizing nature of these networks enables the use of dynamic reconfiguration mechanisms. These mechanisms can supplement or replace failed key nodes without compromising the integrity of other chains, thereby maximizing the recovery of functional chains and overall network performance.

In MANETs, the failure of a node removes it and all its incident links. As failures accumulate, the network transitions from a connected to a fragmented state, reducing the number of complete functional chains and blocking information flow, ultimately causing the network to fail its mission [6]. Node mobility is another critical factor in connectivity reliability [7]. First, rapid movement causes real-time changes in node positions. Under limited communication range, increasing inter-node distances elevate the risk of link disconnection, which invalidates existing routes and disrupts continuous data transmission. Second, mobility-induced topology changes cause dynamic fluctuations in channel conditions (e.g., signal strength and interference). Severe channel degradation increases bit error rates and can lead to intermittent or failed transmissions, compromising overall communication quality and stability.

Conversely, the inherent self-organization and node mobility of these networks also create opportunities for connectivity restoration [3,7]. Self-organization facilitates the continuous establishment of new connections or the re-establishment of broken ones, ensuring information flow. Meanwhile, node mobility allows functional nodes to physically reposition themselves around failed ones, taking over their communication coverage and forming new links. Thus, the self-organizing and adaptive nature of MANETs contributes to a form of “dynamic self-organizing behavior” that strengthens overall connectivity reliability [6,8,9].

Existing metrics fail to adequately capture this dynamically managed nature of network reliability in heterogeneous functional chain networks. The Capacitated Resilience (CR) metric proposed by Kabadurmus and Smith [10], while commendably integrating capacity, path reliability, and rerouting capability, is fundamentally a supply–demand flow model designed for fixed-infrastructure heterogeneous wireless networks (HetNets) at the network design stage. It does not account for node functional role heterogeneity, node mobility, or runtime dynamic reconfiguration. Mission reliability models based on k-out-of-n: F systems [11] assume homogeneous units operating in fixed formations and thus cannot be generalized to heterogeneous functional chain networks. Resilience metrics proposed for Flying Ad Hoc Networks [12] and Unmanned System-of-Systems [13] operate at the macroscopic system or system-of-systems level, assessing aggregate performance trends rather than the micro-level structural integrity of individual functional chains. Table 1 and Table 2, respectively, demonstrate the differences between existing metrics and functional chain connectivity reliability from the perspectives of evaluation objects and evaluation granularity.

Critically, none of these metrics addresses the following core question: given a heterogeneous MANET in which nodes with distinct functional roles are organized into functional chains (e.g., Sensing→Decision→Action, following the OODA loop paradigm), how does the connectivity reliability of these functional chains evolve over time under the joint effects of node failures, node mobility, link unreliability, and dynamic reconfiguration? The aforementioned analysis unveils the core innovative logic of this paper: as networks evolve from “homogeneous interconnection” to “heterogeneous collaboration”, the evaluation paradigm must transition from “topological connectivity” to “functional closure”. The essence of this shift lies in the question from “whether the network can be connected” to “whether each functional chain is complete and whether the network can manage itself to maintain these functional chains”. This reflects the “manageability” of network reliability and constitutes the theoretical contribution of this paper.

For practical engineering application systems, the reliability of a single network node or a single-hop link can be determined through historical data or experiments. However, when heterogeneous nodes and links are combined into different functional chains, and there is a coupling relationship between these functional chains, the network connectivity reliability is no longer a simple function of the reliability or failure rate of network components. Its time-varying characteristics are crucial for network tasks. Compared with existing research, this paper mainly analyzes the impact of dynamic reconfiguration mechanisms on the connectivity reliability of heterogeneous functional chain networks composed of imperfect nodes and imperfect links. Firstly, the paper analyzes the constituent elements of general heterogeneous MANETs, establishes their graph theory models in the order of node–link–network, and models the failure characteristics and mobility characteristics of nodes as well as the reliability of links separately. Secondly, the paper conducts conceptual modeling and related algorithm design for the functional chain grouping rules and dynamic reconfiguration mechanisms in heterogeneous MANETs. Subsequently, the paper proposes evaluation metrics and simulation algorithms for the connectivity reliability of heterogeneous MANETs based on functional chains. Finally, the algorithm implementation and simulation experiment verification are completed based on the MATLAB platform. The main research contributions of this paper are as follows:

Extension of network connectivity reliability evaluation models and methods. Beyond the inherent reliability of individual components (nodes and links), this work extends the analysis to the impact of specific evolutionary processes on system-level reliability. By modeling the dynamic reconfiguration mechanism, it reveals how the self-organizing and self-adaptive nature of MANETs shapes their connectivity reliability.

Construction of a general functional chain network model. Departing from generic task scenarios and network components, a unified heterogeneous functional chain network model is established based on the “perception-decision-action” paradigm. Sub-processes are systematically mapped onto heterogeneous node types according to practical engineering requirements, thereby expanding the applicability of the network model.

Proposal of function- and task-oriented connectivity reliability evaluation metrics and algorithms. Leveraging the flexible deployment and scalable nature of network systems, this work proposes evaluation parameters and simulation-based algorithms for assessing the connectivity reliability of heterogeneous functional chain networks, driven by functional and task-level requirements. These metrics enable cross-comparison across networks of different scales.

## 2. Related Work

This section reviews the literature relevant to the connectivity reliability evaluation of heterogeneous MANETs and is organized into two thematic areas. Section 2.1 surveys methods for reliability evaluation in heterogeneous networks. It begins with a summary of foundational studies on MANET communication reliability and then systematically examines capacity-aware reliability metrics, resilience assessment frameworks for networked systems, structural robustness models, and topology optimization approaches. The common limitation across these methods is identified as their predominant focus on static or quasi-static network properties without accounting for runtime reconfigurability. Section 2.2 examines dynamic reconfiguration mechanisms in MANETs, covering mission reliability optimization through importance measures, resilience analysis incorporating spatio-temporal network dynamics, and cooperative reconfiguration strategies at the system-of-systems level. Collectively, the reviewed works highlight a gap: existing reliability metrics in heterogeneous networks are assessed either as intrinsic static properties or as post-failure recovery outcomes, whereas the connectivity reliability of functional chains under continuous runtime reconfiguration remains underexplored—motivating the methodology developed in this paper.

### 2.1. Reliability Evaluation for Heterogeneous Networks

Recent advances in the reliability assessment of MANETs have taken diverse modeling approaches across different application domains. Zhong et al. [1] addressed GPS spoofing attacks in UAV networks by proposing a Byzantine Distributed GPS Detection (BDGD) algorithm that integrates received signal strength indicator (RSSI) ranging verification with adaptive reputation scoring, achieving distributed malicious-node detection and isolation without global synchronization. Wang et al. [6] investigated the disintegration of heterogeneous UAV swarms under communication constraints. At the reliability modeling level, they constructed a self-organizing reconfiguration model incorporating node connectivity and channel capacity, and at the reliability evaluation level, they proposed a task-oriented network resilience metric system based on percolation theory with connectivity robustness (CCR) and information transfer efficiency (IER) as core indicators. Ali et al. [14] conducted a comprehensive survey on UAV-assisted device-to-device (D2D) communication across seven heterogeneous network domains and systematically analyzed the mechanisms through which transmission power, interference mitigation, and channel dynamics affect D2D link reliability. Zhuang et al. [3] developed a network recovery scheme with heterogeneous graph neural networks (NRHG) for UAV-assisted IoTs after cascading failures, leveraging HGNN for local observation processing and multi-agent reinforcement learning for coordinated recovery decisions. Wang and Tian et al. [15] proposed a transmission reliability evaluation model for military MANETs that jointly considers random failures, energy-consumption failures, and channel capacity randomness using the sum of disjoint products (SDP) method and Monte Carlo simulation. While these studies collectively demonstrate the breadth of MANET reliability research, a common thread emerges: reliability is typically assessed either as a static network property or as a post-disruption recovery outcome. The dynamic interplay between runtime reconfiguration and functional chain integrity—where individual chains continuously degrade and self-repair through node-level replacement—has not been systematically addressed, motivating the functional-chain-oriented evaluation framework proposed in this paper.

Kabadurmus et al. [10] addressed the issue in traditional reliability/survivability metrics (such as K-terminal reliability, full-terminal reliability, and traffic efficiency) in telecommunications network design, which generally overlook link and device capacity and fail to fully consider the requirements for rerouting and traffic offloading. In light of the development background of heterogeneous wireless networks (HetNets) catalyzed by the integration of technologies such as 4G and WiFi, they proposed a new metric that integrates network reliability and resilience—Capacitated Resilience (CR). CR can simultaneously consider capacity constraints, path reliability, rerouting capability, and traffic offloading, distinguishing between user-level and network-level metrics. Its value ranges from 0 to 1, facilitating comparison across different network designs. A comprehensive review by Murphy et al. [16] examined machine learning applications for fault prediction in heterogeneous telecommunications networks, highlighting the gap between conventional reliability assessments and industrial operational constraints. Zhong et al. [17] focused on the resilience measurement and optimization research of the networked command information system, which consists of four types of nodes: intelligence acquisition node, information processing node, decision-making node, and action execution node.

For heterogeneous computing networks, a hypergraph-based resource model and a collaborative task offloading optimization framework were developed in [18] leveraging hypergraph neural networks and particle swarm optimization to enhance reliability under node failures and link instability. Qian et al. [19] focused on the robustness of “One-to-Many Interdependent Higher-Order Networks” against cascading failures under random attacks and constructed a network model incorporating intra-layer high-order interactions and one-to-many inter-layer dependencies. Through validation using homogeneous and heterogeneous synthetic supergraphs and real datasets from high- and medium-contact networks, they revealed the impact of intra-layer high-order structure and coupling strength on robustness. Lin et al. [20] proposed the DSR-VFPSO algorithm to enhance the resilience of deep-sea acoustic sensor networks by optimizing both coverage and betweenness centrality under a fully connected sink-node constraint. The core feature of the sink-node network model is the deployment of a centralized sink node, which serves as the data aggregation and processing hub for the entire network. The remaining nodes in the network are general-purpose sensor nodes. Liu et al. [21] focused on in-network computing (INC) services as their research subject. Addressing the shortcomings of existing reliable service deployments that only consider software reliability while neglecting hardware heterogeneity reliability, which leads to service interruptions and high costs, they proposed a deployment scheme that integrates hardware and software reliability modeling with cost provability. They defined the problem of INC-enabled Services Adoption with Heterogeneous Reliability (ISAHR) and proved its NP-hardness.

Wang et al. [6] developed an adaptive disintegration strategy (ADHGR) for heterogeneous UAV swarms using graph reinforcement learning to optimize node removal decisions under communication constraints. Ali et al. [22] systematically investigated UAV-assisted Device-to-Device (D2D) communication across seven heterogeneous network domains, covering key technologies such as power allocation, interference suppression, and blockchain authentication, and identified future directions including 6G integration and AI-driven UAV cluster management. Zhuang et al. [3] focused on unmanned aerial vehicle (UAV)-assisted Internet of Things (IoT) as the research subject. Addressing the challenges of topological integrity disruption, network coverage, and system throughput recovery in heterogeneous networks comprising satellite UAVs (SUAVs), ordinary UAVs (OUAVs), and fixedly deployed IoT devices after cascading failures, they proposed a network recovery scheme based on Heterogeneous Graph Neural Network (NRHG).

### 2.2. Dynamic Reconfiguration Mechanisms in MANETs

Dui et al. [11] conducted research on the task reliability and structural optimization of polygonal formation (such as triangular and quadrilateral) drone swarms. They modeled the task reliability of polygonal formation UAV swarms by fitting them into a continuous k-out-of-n: F system and applying the finite Markov chain embedding method. Key drones were identified using Birnbaum and Integrated Importance Measures, and optimization strategies combining conditional reliability and remaining useful life were developed for preventive maintenance and post-failure relocation. Ma et al. [23] proposed a belief resilience metric based on uncertainty theory for low-Earth-orbit satellite communication systems, integrating both aleatory and epistemic uncertainties. A dynamic satellite network evolution model was constructed to characterize topological dynamics, and the complete evaluation framework was validated on a Walker constellation. Guo et al. [12] proposed an improved resilience analysis method for Flying Ad Hoc Networks (FANETs) in conflict environments, integrating spatio-temporal network dynamics with stochastic geometry theory to capture the impact of time-varying topologies on collaborative missions. The resilience of Unmanned System-of-Systems (USoS) was addressed in [13] through a three-layer “task-capability-resource” framework, where collaborative reconfiguration strategies were shown to significantly improve performance under targeted attacks.

The literature reviewed above suggests that research on network reliability can be broadly classified into two categories. The first concerns a priori reliability [24], which examines how intrinsic network properties—such as the size of the largest connected component, network coverage, and network lifespan—degrade following node and link failures. The second concerns a posteriori reliability [4], which captures the dynamic reliability patterns that emerge from self-organization, self-adaptation, and reconfiguration after failures, reflected in metrics such as the number of intact functional chains, robustness, and invulnerability. Focusing on the latter, this paper considers heterogeneous MANETs under the combined effects of internal/external disruptions (e.g., node failures and intentional attacks) and link communication quality and investigates the evolution of connectivity reliability in MANETs equipped with dynamic reconfiguration mechanisms. Table 3 reveals a clear gap in the literature: no existing metric simultaneously satisfies all four requirements that characterize the problem addressed in this work—(1) functional chain as the assessment object, (2) node functional role heterogeneity, (3) dynamic reconfiguration as a runtime mechanism, and (4) continuous time-domain evolution analysis.

## 3. Modeling of Heterogeneous Functional Chain MANETs

### 3.1. Network Structure and Functional Element Classification

This paper adopts a heterogeneous directed graph model [26] to characterize MANETs, formulated as $G=\left(V,E\right)$. Here, G denotes the graph-theoretic model of the MANET, $V=\left\{N_{1},N_{2},\ldots ,N_{m}\right\}$ is the node set, and $E=\left\{e_{ij}|i\neq j\right\}$ represents the edge set, where $e_{ij}=\left\{\left(N_{i},N_{j}\right)|i\neq j\right\}$ denotes the directed link from source node $N_{i}$ to sink node $N_{j}$. Node and link heterogeneity are characterized by mapping functions $\varphi :V\to V_{type}$ and $\psi :E\to E_{type}$, respectively, where $V_{type}$ and $E_{type}$ are predefined node and edge types satisfying $\left|V_{type}\right|\geq 2$, $\left|E_{type}\right|\geq 2$, and $\max\left|E_{type}\right|={\left|V_{type}\right|}^{2}$.

Following the typical task execution flow of MANETs [17], the network is abstracted into a four-stage closed-loop cycle: environment sensing, task allocation, node execution, and environment re-sensing. Correspondingly, network elements are divided into three categories: reconnaissance-sensing elements, command-decision elements, and action-execution elements.

#### 3.1.1. Reconnaissance-Sensing Elements

The reconnaissance-sensing elements primarily refer to various sensors and platforms deployed on MANET nodes, including satellites, drones, radars, high-resolution cameras, and other equipment. These elements are responsible for environmental situational awareness, task target identification, multi-source intelligence information acquisition, and information processing and transmission. In MANETs, multiple reconnaissance-sensing elements or systems may be deployed simultaneously on the same node to work together. Furthermore, reconnaissance and detection systems between different nodes can also cooperate with each other. By optimizing the deployment and working modes of detection resources, the detection efficiency of MANETs can be improved.

#### 3.1.2. Command-Decision Elements

The command-decision element, as the core of MANETs, primarily refers to various control devices and decision support systems deployed on network nodes. After receiving and processing data from the reconnaissance-sensing elements, the command-decision element further fulfills responsibilities such as situation visualization and judgment, action planning and task allocation plan generation, as well as control command issuance. During this process, by integrating AI and other technical elements, network commanders can achieve fine-tuning and optimization of action plans. Furthermore, in MANETs, there often exists one or a few command-decision elements (systems) as the highest-level control platform, responsible for issuing subtask commands to all network nodes and leading all nodes to achieve the overall network task. Secondary command-decision elements, upon receiving subtask information, are responsible for processing and executing it, ultimately forming a hierarchical phenomenon of control relationships within the MANET.

#### 3.1.3. Action-Execution Elements

As the ultimate execution unit in MANETs, the action-execution element is crucial for implementing specific tasks. It primarily refers to various action platforms equipped on network nodes, including firefighting equipment, transportation and delivery equipment, or excavation equipment. In a highly dynamic, multi-temporal, uncertain, and complex task environment, different MANETs are equipped with different action platforms with varying capabilities. Based on the location and capability status of the network nodes, each action-execution element autonomously collaborates to complete various tasks after receiving instructions from the command-decision element. The action-execution element and its capability are considered as one of the key factors affecting the functionality of MANETs.

Based on the above classification, heterogeneous nodes are divided into three types: decision nodes $N^{C}=\left\{N_{c}^{C}|c=1,2,\ldots ,m_{1}\right\}$, sensor nodes $N^{S}=\left\{N_{s}^{S}|s=1,2,\ldots ,m_{2}\right\}$, and action execution nodes $N^{W}=\left\{N_{w}^{W}|w=1,2,\ldots ,m_{3}\right\}$, where $m=m_{1}+m_{2}+m_{3}$ and $\left|V_{type}\right|=3$. Each node is identified by both a global index i and a type-specific ordinal (s,c,w), with the mapping relationship given by

(1) $$\left\{\begin{array}{l} N_{c}^{C}=N_{c},\;c=1,2,\ldots ,m_{1}\\ N_{s}^{S}=N_{s+m_{1}},\;s=1,2,\ldots ,m_{2}\\ N_{w}^{W}=N_{w+m_{1}+m_{2}},\;w=1,2,\ldots ,m_{3} \end{array}\right.$$

### 3.2. Node Modeling: Failure Modes and Mobility Characteristics

Heterogeneous nodes are susceptible to failures induced by internal and external disturbances during operation. Such failures directly impair node functionalities, disconnect links, disrupt functional chains, and degrade network connectivity reliability. Meanwhile, node mobility (varying speed, direction, acceleration) changes real-time positions and Euclidean distances, further affecting link quality and network topology. This section models node failure modes and mobility behavior, as well as link reliability.

This paper defines two functional failure modes and one structural failure mode: hardware/software failure, intentional attack-induced failure, and isolation failure. Functional failure refers to the mode in which a network node fails to perform its designated function normally, thus being determined as faulty. Hardware/software failure is used to characterize sporadic failures of network nodes and failures caused by factors such as manufacturing deviations. Considering the applicability of exponential distribution in classical reliability theory, the paper uses an exponential distribution model to model hardware/software failures of network nodes [27]. Intentional attack is used to characterize node failures caused by external attacks when heterogeneous mobile ad hoc networks perform specific tasks. For example, drones close to the fire source in fire-fighting tasks are more likely to be damaged by factors such as local explosions, high temperatures, and falling building debris. Vehicle-to-everything (V2X) nodes are more susceptible to attacks such as Distributed Denial of Service (DDoS) when approaching unsafe Road Side Units (RSU). The paper assumes that the probability of a network node failing due to intentional attack depends on the Euclidean distance between the node and the hazard source. Isolation failure is used to characterize the failure of a functionally intact node that cannot establish a single-hop connection with other nodes in the network and cannot be incorporated into the network topology. For example, a node may move out of the communication coverage range of its neighbors, or the original neighbors of a node may fail, causing it to no longer have communicable neighbors. Four node operational states are formally defined: $S_{N_{i}}=1$ denotes a fault-free node operating normally in the network; $S_{N_{i}}=2$ denotes a node with hardware/software failure; $S_{N_{i}}=3$ denotes a node that has failed due to intentional attack; and $S_{N_{i}}=4$ denotes a node with isolation failure.

Let $\lambda_{i}$ denote the constant failure rate of node $N_{i}$ due to hardware/software malfunctions. The probability that node $N_{i}$ fails in this mode by time t follows the exponential distribution

(2) $$\mathrm{Pr}\left(S_{N_{i}}\left(t\right)=2\right)=1-e^{-\lambda_{i}t}$$

Let $d_{iTa}$ denote the Euclidean distance between node $N_{i}$ and the hazard source. The paper simplifies the analysis of attack events based on relative distance and hazard source coverage, referencing existing literature research [28,29,30,31,32], and the intentional attack failure event of the node is modeled as an exponential distribution, where the parameter $\lambda_{i}^{T}$ is inversely proportional to $d_{iTa}$. Then, the probability of the node $N_{i}$ failing due to intentional attack at time t is

(3) $$\left\{\begin{array}{l} \mathrm{Pr}\left(S_{N_{i}}\left(t\right)=3\right)=1-e^{-\lambda_{i}^{T}t}\\ \lambda_{i}^{T}=\frac{1}{d_{iTa}} \end{array}\right.$$

**Figure 1:**
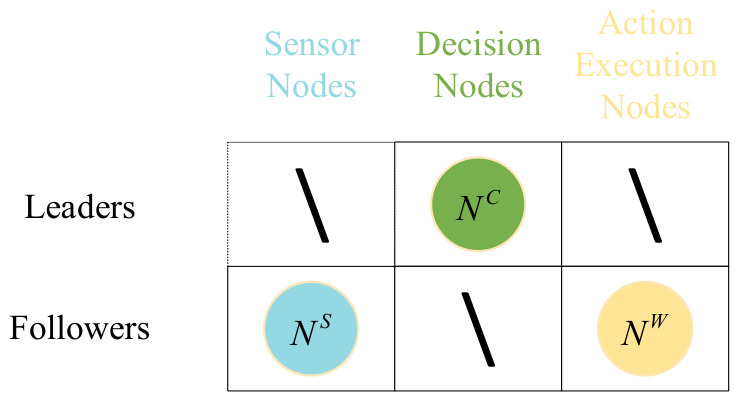
Correspondence between node types in MANETs and roles in the Couzin-leader model.

Based on the Couzin-leader model [33,34,35,36], from a motion perspective, network nodes can be divided into two key roles: leaders and followers. There are four motion rules within the network: repulsion, attraction, alignment, and homing. Leaders grasp the overall network and task information, exert influence on the follower nodes in the network to change their behavior, and guide the follower nodes to jointly complete the network tasks. In heterogeneous MANETs, the decision nodes $N^{C}=\left\{N_{c}^{C}|c=1,2,\ldots ,m_{1}\right\}$ and leader nodes are role-matched, and sensor nodes $N^{S}=\left\{N_{s}^{S}|s=1,2,\ldots ,m_{2}\right\}$, action execution nodes $N^{W}=\left\{N_{w}^{W}|w=1,2,\ldots ,m_{3}\right\}$ and followers are role-matched. The correspondence between the types of nodes in MANETs and the roles in the Couzin-leader model is illustrated in Figure 1.

The repulsion rule refers to the maintenance of a certain safe distance $d_{Co}$ between nodes in heterogeneous MANETs to avoid collisions and reduce mutual communication interference. The attraction rule refers to the attraction of neighboring nodes that meet certain conditions between nodes in heterogeneous MANETs to avoid network splitting, with the attraction range being a circular plane with a radius of $\left(d_{Co},d_{At}\right]$. The alignment rule refers to the adjustment of the direction angle $\theta_{i}$ of motion between nodes in heterogeneous MANETs, making them face the average direction of neighboring nodes and thereby manifesting as a common forward direction towards the overall network. The homing rule refers to the need for leaders, i.e., decision-making nodes, in the network to lead the overall network towards a predetermined direction (such as a task target point). This paper improves the Couzin-leader model based on these four motion rules to make it conform to the deployment and operational reality of heterogeneous networks. Let ${\hat{O}}_{i}^{re}$, ${\hat{O}}_{i}^{at}$, ${\hat{O}}_{i}^{al}$ and ${\hat{O}}_{i}^{ho}$ be the repulsion direction vector, attraction direction vector, alignment direction vector, and homing direction vector of node $N_{i}$, respectively. Let $d_{ij}$ be the Euclidean distance between node $N_{i}$ and $N_{j}$.

The set of neighbor nodes $Nei^{d_{Co}}$ that the node $N_{i}$ needs to exclude and the exclusion direction vector ${\hat{O}}_{i}^{re}$ can be represented as

(4) $$Nei^{d_{Co}}=\left\{N_{j}|d_{ij}\leq d_{Co},j\neq i\right\}$$

(5) $$\begin{array}{ll} O_{i}^{re}\left(t+\Delta t\right) & =\left(\cos\theta_{i}\left(t+\Delta t\right),\sin\theta_{i}\left(t+\Delta t\right)\right)\\ & =-\sum_{N_{j}\in Nei^{d_{Co}}}\frac{\left(x_{j}\left(t\right),y_{j}\left(t\right)\right)-\left(x_{i}\left(t\right),y_{i}\left(t\right)\right)}{\left\|\left(x_{j}\left(t\right),y_{j}\left(t\right)\right)-\left(x_{i}\left(t\right),y_{i}\left(t\right)\right)\right\|} \end{array}$$

(6) $${\hat{O}}_{i}^{re}\left(t+\Delta t\right)=\frac{O_{i}^{re}\left(t+\Delta t\right)}{\left\|O_{i}^{re}\left(t+\Delta t\right)\right\|}$$

In the formula, $\left\|O_{i}^{re}\left(t+\Delta t\right)\right\|$ represents the norm of the vector $O_{i}^{re}\left(t+\Delta t\right)$, and the repulsive unit vector ${\hat{O}}_{i}^{re}\left(t+\Delta t\right)$ of node $N_{i}$ is obtained via normalization.

For node $N_{i}$, it will first attract neighboring nodes $Nei_{1}^{d_{At}}$ that belong to the same functional chain $l_{j}^{kc}$, and then generate attraction towards the remaining set of neighboring nodes $Nei_{2}^{d_{At}}$ within the circular plane with a radius of $\left(d_{Co},d_{At}\right]$. The set of neighboring nodes it attracts, as well as the attraction direction vector ${\hat{O}}_{i}^{at}$, can be expressed as follows:

(7) $$Nei_{1}^{d_{At}}=\left\{N_{u}|N_{u}\in l_{j}^{kc},u\neq i\right\}$$

(8) $$Nei_{2}^{d_{At}}=\left\{N_{j}|d_{Co}<d_{ij}\leq d_{At},j\neq i,N_{j}\notin l_{j}^{kc}\right\}$$

(9) $$\begin{array}{ll} O_{i}^{at}\left(t+\Delta t\right) & =\omega_{1}^{d_{At}}\sum_{N_{u}\in Nei_{1}^{d_{At}}}\frac{\left(x_{u}\left(t\right),y_{u}\left(t\right)\right)-\left(x_{i}\left(t\right),y_{i}\left(t\right)\right)}{\left\|\left(x_{u}\left(t\right),y_{u}\left(t\right)\right)-\left(x_{i}\left(t\right),y_{i}\left(t\right)\right)\right\|}\\ & +\omega_{2}^{d_{At}}\sum_{N_{j}\in Nei_{2}^{d_{At}}}\frac{\left(x_{j}\left(t\right),y_{j}\left(t\right)\right)-\left(x_{i}\left(t\right),y_{i}\left(t\right)\right)}{\left\|\left(x_{j}\left(t\right),y_{j}\left(t\right)\right)-\left(x_{i}\left(t\right),y_{i}\left(t\right)\right)\right\|} \end{array}$$

(10) $${\hat{O}}_{i}^{at}\left(t+\Delta t\right)=\frac{O_{i}^{at}\left(t+\Delta t\right)}{\left\|O_{i}^{at}\left(t+\Delta t\right)\right\|}$$

In the formula, $\omega_{1}^{d_{At}}$ and $\omega_{2}^{d_{At}}$ represent the attraction weights of node pairs with and without neighboring nodes in the same functional chain, respectively.

Node $N_{i}$ prioritizes alignment with neighboring nodes $Nei_{1}^{d_{At}}$ within the same functional chain, followed by alignment with neighboring nodes $Nei_{2}^{d_{At}}$ not within the same functional chain. The calculation formula for the node alignment direction vector ${\hat{O}}_{i}^{al}$ is as follows:

(11) $$O_{i}^{al}\left(t+\Delta t\right)=\frac{O_{i}\left(t\right)}{\left\|O_{i}\left(t\right)\right\|}+\sum_{N_{u}\in Nei_{1}^{d_{At}}}\frac{O_{u}\left(t\right)}{\left\|O_{u}\left(t\right)\right\|}+\sum_{N_{j}\in Nei_{2}^{d_{At}}}\frac{O_{j}\left(t\right)}{\left\|O_{j}\left(t\right)\right\|}$$

(12) $${\hat{O}}_{i}^{al}\left(t+\Delta t\right)=\frac{O_{i}^{al}\left(t+\Delta t\right)}{\left\|O_{i}^{al}\left(t+\Delta t\right)\right\|}$$

The formula for calculating the direction vector of node $N_{i}$ is

(13) $$O_{i}^{\prime }\left(t+\Delta t\right)=\frac{{\hat{O}}_{i}^{re}\left(t+\Delta t\right)+{\hat{O}}_{i}^{at}\left(t+\Delta t\right)+{\hat{O}}_{i}^{al}\left(t+\Delta t\right)}{\left\|{\hat{O}}_{i}^{re}\left(t+\Delta t\right)+{\hat{O}}_{i}^{at}\left(t+\Delta t\right)+{\hat{O}}_{i}^{al}\left(t+\Delta t\right)\right\|}$$

(14) $${\hat{O}}_{i}^{ho}\left(t+\Delta t\right)=\frac{O_{i}^{\prime }\left(t+\Delta t\right)+\omega_{i}g}{\left\|O_{i}^{\prime }\left(t+\Delta t\right)+\omega_{i}g\right\|}$$

In the formula, g represents a unit vector, indicating the overall task information of the heterogeneous mobile ad hoc network; $\omega_{i}$ is a weight term, with $\omega_{i}=0$ indicating that the node has no understanding of the overall task information of the heterogeneous mobile ad hoc network and moves entirely under the influence of neighboring nodes; $\omega_{i}=1$ represents that the node has partial understanding of the overall task information of the heterogeneous mobile ad hoc network and is influenced by both the overall task information and neighboring nodes; and $\omega_{i}>1$ indicates that the node prefers to complete the overall task of the heterogeneous mobile ad hoc network by influencing the movement of neighboring nodes.

In summary, the updated direction vector $O_{i}\left(t+\Delta t\right)$ of the heterogeneous mobile ad hoc network node $N_{i}$ at time t+Δt can be obtained:

(15) $$O_{i}\left(t+\Delta t\right)=\frac{{\hat{O}}_{i}^{re}\left(t+\Delta t\right)+{\hat{O}}_{i}^{at}\left(t+\Delta t\right)+{\hat{O}}_{i}^{al}\left(t+\Delta t\right)+{\hat{O}}_{i}^{ho}\left(t+\Delta t\right)}{\left\|{\hat{O}}_{i}^{re}\left(t+\Delta t\right)+{\hat{O}}_{i}^{at}\left(t+\Delta t\right)+{\hat{O}}_{i}^{al}\left(t+\Delta t\right)+{\hat{O}}_{i}^{ho}\left(t+\Delta t\right)\right\|}$$

### 3.3. Link Reliability Modeling

The FS-TRG model [37] is utilized to characterize link reliability between heterogeneous nodes, which is mathematically defined as

(16) $$R_{e}\left(d_{ij}\left(t\right)\right)=\left\{\begin{array}{ll} 1, & d_{ij}\left(t\right)\leq \alpha d_{Th}\\ \left(\frac{\alpha^{2}}{1-\alpha^{2}}\right)\left(\frac{d_{Th}^{2}}{d_{ij}^{2}\left(t\right)}-1\right), & \alpha d_{Th}\leq d_{ij}\left(t\right)\leq \beta d_{Th}\\ \left(\frac{\alpha^{2}\beta^{2}}{\left(1-\alpha^{2}\right)\left(1+\beta^{2}\right)}\right)\left(\frac{d_{Th}^{4}}{d_{ij}^{4}\left(t\right)}-1\right), & \beta d_{Th}\leq d_{ij}\left(t\right)\leq d_{Th}\\ 0, & d_{ij}\left(t\right)\geq d_{Th} \end{array}\right.$$

where $R_{e}\left(d_{ij}\left(t\right)\right)$ is the reliability of link $e_{ij}$ between nodes $N_{i}$ and $N_{j}$ at time t; $d_{Th}$ is the communication distance threshold; 0<α<β<1 are the propagation model parameters. For short-range ($d_{ij}\left(t\right)\leq \alpha d_{Th}$) links, $R_{e}\left(d_{ij}\left(t\right)\right)$ is always equal to 1; for medium-range ($\alpha d_{Th}\leq d_{ij}\left(t\right)\leq \beta d_{Th}$) links, $R_{e}\left(d_{ij}\left(t\right)\right)$ is inversely proportional to $d_{ij}^{2}\left(t\right)$; for long-range ($\beta d_{Th}\leq d_{ij}\left(t\right)\leq d_{Th}$) links, $R_{e}\left(d_{ij}\left(t\right)\right)$ is inversely proportional to $d_{ij}^{4}\left(t\right)$; and for links exceeding the threshold ($d_{Th}<d_{ij}\left(t\right)$), $R_{e}\left(d_{ij}\left(t\right)\right)$ is always equal to 0. To quantify reliable communication links, a link reliability threshold $R_{e}^{Th}$ is set to judge the link state: if $R_{e}\left(d_{ij}\left(t\right)\right)\geq R_{e}^{Th}$, it is determined that there is a reliable single-hop link $e_{ij}$ between nodes $N_{i}$ and $N_{j}$ at time t; otherwise, it is determined that there is no single-hop link between nodes $N_{i}$ and $N_{j}$. Based on the above link state criterion, the binary adjacency matrix $A_{RB}\left(t\right)$ of the heterogeneous MANET is constructed as

(17) $$A_{RB}\left(t\right)=\left[\begin{array}{cccc} a_{RB11}\left(t\right) & a_{RB12}\left(t\right) & \ldots & a_{RB1m}\left(t\right)\\ a_{RB21}\left(t\right) & a_{RB22}\left(t\right) & \ldots & a_{RB2m}\left(t\right)\\ \vdots & \vdots & \ddots & \vdots \\ a_{RBm1}\left(t\right) & a_{RBm2}\left(t\right) & \ldots & a_{RBmm}\left(t\right) \end{array}\right]$$

(18) $$a_{RBij}\left(t\right)=\left\{\begin{array}{l} 1,\;R_{e}\left(d_{ij}\left(t\right)\right)\geq R_{e}^{Th}\\ 0,\;else \end{array}\right.$$

In this formulation, $a_{RBij}\left(t\right)$ serves as an indicator of the link state between nodes. If node $N_{i}$ fails in the heterogeneous MANET at time t, all links between the node $N_{i}$ and its neighboring nodes before time t will be disconnected, and the corresponding elements in the adjacency matrix $A_{RB}\left(t\right)$ will be updated:

(19) $$\left\{\begin{array}{l} a_{RBij}\left(t\right)=0,\;i\neq j\\ a_{RBji}\left(t\right)=0,\;j\neq i \end{array}\right.$$

For a normal working node $N_{i}$, if some links are disconnected due to the failure of neighboring nodes, or if it moves out of the communication coverage range of neighboring nodes, resulting in the corresponding element in the adjacency matrix $A_{RB}\left(t\right)$ satisfying the condition $\sum a_{RBij}\left(t\right)=\sum a_{RBji}\left(t\right)=0$ (j∈V,j≠i), it is determined that the node $N_{i}$ has experienced isolation failure at time t.

**Figure 2:**
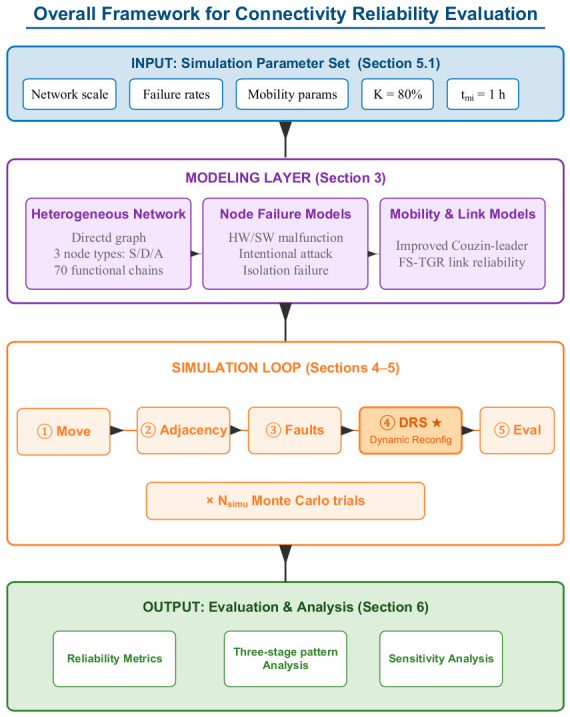
Overall framework of the proposed connectivity reliability evaluation methodology for heterogeneous functional chain networks with dynamic reconfiguration.

To provide a synoptic view of the complete methodology before proceeding to the algorithmic details, Figure 2 presents the overall framework of the proposed evaluation approach. The framework integrates the four modeling components established in Section 3—heterogeneous functional chain graph, multi-mode node failure, improved Couzin-leader mobility model, and FS-TRG link reliability model—into an iterative Monte Carlo simulation loop, with the Dynamic Reconfiguration Scheme (DRS) serving as the central runtime intervention mechanism. The subsequent sections elaborate on the DRS algorithm (Section 4), the full simulation procedure (Section 5), and the case study results obtained from this framework (Section 6).

## 4. Functional Chain Formation and Dynamic Reconfiguration Mechanisms

### 4.1. Functional Chain Structure and Initial Formation

Building on the node failure model, mobility model, and link reliability model established in Section 3, this section defines the core functional unit of our heterogeneous MANET—the end-to-end functional chain—and describes the initial fixed-pairing formation rule that serves as the baseline for connectivity reliability evaluation.

This study quantifies the connectivity reliability of heterogeneous MANETs based on Boyd’s OODA loop theory [38], which aligns perfectly with the sensing–decision–action execution cycle of heterogeneous functional chain networks. The overall mission performance of these networks depends on the collective operation of multiple independent functional chains, each executing a complete end-to-end task. In conjunction with practical scenarios, three criteria governing functional chain impacts on MANET performance are proposed: (1) shorter chain length improves information transmission accuracy/timeliness and task execution capability; (2) a larger number of functional chains enhances mission success probability and overall efficiency; and (3) more functional chains correspond to higher task scheme diversity and stronger network reliability and robustness.

**Figure 3:**
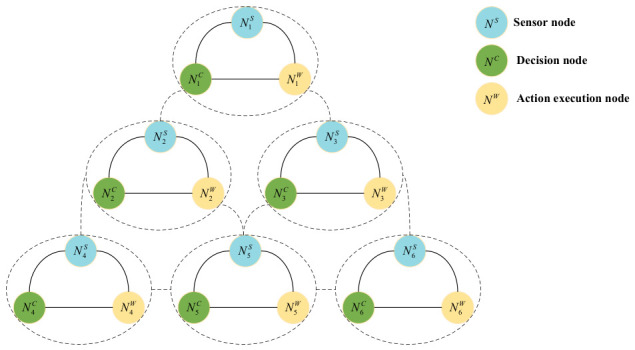
Fixed-formation heterogeneous mobile ad hoc network.

Let $l_{j}^{kc}$ denote a functional chain composed of sensor nodes $N^{S}$, decision nodes $N^{C}$, and action execution nodes $N^{W}$, which follows the sensing–decision–action paradigm with three serially interdependent links. Failure of any single link invalidates the entire chain, as it cannot fulfill its intended function. Thus, the functionality of a chain depends entirely on the collaborative operation of all three node types. A fixed-pairing heterogeneous functional chain network (denoted as $G_{fd}$) is adopted, and its structure is illustrated in Figure 3. As shown in Figure 3, dedicated nodes form functional chains via wireless links, e.g., $l_{1}^{kc}=\left\{N_{1}^{S},N_{1}^{C},N_{1}^{W}\right\}$ and $l_{2}^{kc}=\left\{N_{2}^{S},N_{2}^{C},N_{2}^{W}\right\}$. Each node $N_{i}$ (i=1,2,…,m) belongs to exactly one chain $l_{j}^{kc}$ (j=1,2,…,KC). Failure of any node in $l_{j}^{kc}=\left\{N_{j}^{S},N_{j}^{C},N_{j}^{W}\right\}$ renders the entire chain invalid. Distinct chains interact via cooperative sensing, distributed decision-making, and coordinated execution.

### 4.2. Dynamic Reconfiguration Principles and Workflow

**Figure 4:**
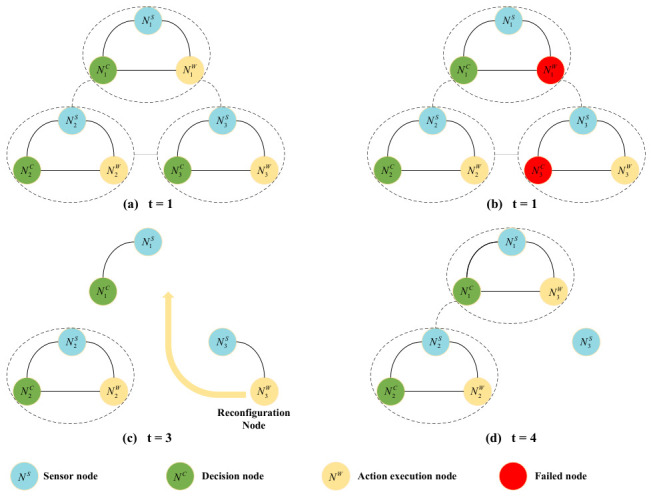
Dynamic reconfiguration process of heterogeneous MANETs. (**a**) Initial network; (**b**) two nodes belonging to different functional chains fail; (**c**) select nodes for dynamic reconfiguration; (**d**) network dynamic reconfiguration is completed.

While the initial fixed-pairing functional chain structure ensures clear task division and low initial deployment complexity, it is inherently vulnerable to node and link failures: a single node outage can render an entire chain non-functional, severely degrading network mission performance. MANETs demonstrate inherent self-organizing and self-adaptive capabilities. During real-world operation, dynamic reconfiguration occurs across functional chains, as depicted in Figure 4. Consider a fixed-formation MANET $G_{fd}$ with three complete chains at t=1: $l_{1}^{kc}=\left\{N_{1}^{S},N_{1}^{C},N_{1}^{W}\right\}$, $l_{2}^{kc}=\left\{N_{2}^{S},N_{2}^{C},N_{2}^{W}\right\}$, $l_{3}^{kc}=\left\{N_{3}^{S},N_{3}^{C},N_{3}^{W}\right\}$, where $N_{kc}\left(1\right)=3$. At t=2 action execution node $N_{1}^{W}$ (in $l_{1}^{kc}$) and decision node $N_{3}^{C}$ (in $l_{3}^{kc}$) fail due to node faults or adversarial attacks. At t=3, removing failed nodes and their links leaves only one complete chain $l_{2}^{kc}$ in $G_{fd}$, so $N_{kc}\left(3\right)=1$. The original functional chains $l_{1}^{kc}$ and $l_{3}^{kc}$ become incomplete and are marked as failed chains $l_{1,F}^{kc}$ and $l_{3,F}^{kc}$. If sensor node $N_{1}^{S}$ and decision node $N_{1}^{C}$ are taken as the basis, the action execution node $N_{3}^{W}$ is selected as the reconfiguration node, and the failed node $N_{1}^{W}$ is replaced. At t=4, action execution node $N_{3}^{W}$ is repositioned, and edges are established between $N_{3}^{W}–N_{1}^{S}$ and $N_{3}^{W}–N_{1}^{C}$, reconstructing a complete chain. The original failed functional chain $l_{1,F}^{kc}$ is reclassified as $l_{1}^{kc}=\left\{N_{1}^{S},N_{1}^{C},N_{3}^{W}\right\}$. The fixed-formation MANET $G_{fd}$ completes one dynamic reconfiguration, increasing the chain count to $N_{kc}\left(4\right)=2$ and enhancing network adaptability and mission success probability in dynamic environments [5].

For the general case, when some nodes in the functional chain $l_{j}^{kc}=\left\{N_{j}^{S},N_{j}^{C},N_{j}^{W}\right\}$ fail, resulting in the disconnection of the functional chain, the functional chain $l_{j}^{kc}$ is marked as a non-complete functional chain or a failed functional chain $l_{j,F}^{kc}$, and $l_{F}^{kc}=l_{F}^{kc}\cup \left\{l_{j,F}^{kc}\right\}$ is updated, where $l_{F}^{kc}=\left\{l_{j,F}^{kc}|j=1,2,\ldots \right\}$ represents the set of all failed functional chains within the heterogeneous mobile ad hoc network at the same time. The nodes that can still work normally in the failed functional chain $l_{j,F}^{kc}$ are marked as candidate nodes, and $V_{Cd}=V_{Cd}\cup \left\{N_{i}\right\}$ is updated, where $V_{Cd}=\left\{N_{i}|N_{i}\in l_{j,F}^{kc},\;S_{N_{i}}=1,\;i=1,2,\ldots ,\;j=1,2,\ldots \right\}$ represents the set of all candidate nodes within the heterogeneous mobile ad hoc network at the same time.

Taking Figure 4 as an example, at the initial moment t=1 (initial stage), $N_{kc}\left(1\right)=3$, $l_{F}^{kc}=\emptyset$, $V_{Cd}=\emptyset$;

At t=2 (node failure stage) and t=3 (dynamic reconfiguration stage), $N_{kc}\left(2\right)=N_{kc}\left(3\right)=1$, $l_{F}^{kc}=l_{F}^{kc}\cup \left\{l_{1,F}^{kc}\right\}\cup \left\{l_{3,F}^{kc}\right\}=\left\{l_{1,F}^{kc},l_{3,F}^{kc}\right\}$, $V_{Cd}=V_{Cd}\cup \left\{N_{1}^{S},N_{1}^{C}\right\}\cup \left\{N_{3}^{S},N_{3}^{W}\right\}=\left\{N_{1}^{S},N_{1}^{C},N_{3}^{S},N_{3}^{W}\right\}$;

At t=4 (dynamic reconfiguration completion stage), $N_{kc}\left(4\right)=2$, $l_{F}^{kc}=l_{F}^{kc}\backslash \left\{l_{1,F}^{kc}\right\}=\left\{l_{3,F}^{kc}\right\}$, $V_{Cd}=V_{Cd}\backslash \left\{N_{1}^{S},N_{1}^{C},N_{3}^{W}\right\}=\left\{N_{3}^{S}\right\}$.

The dynamic reconfiguration evolution of the aforementioned network is essentially a process in which the network dynamically reallocates available functional nodes and rewires inter-node connections, changes the network topology, and alters the connection and coupling relationships between nodes, based on the constantly changing environmental and task requirements, node and link states, and the quantity and integrity of functional chains under the premise of limited resources. The dynamic reconfiguration of the network continuously utilizes the normal working nodes in the candidate node set $V_{Cd}$ to reorganize incomplete functional chains, with the ultimate goal of minimizing the number of incomplete functional chains in the failed functional chain set $l_{F}^{kc}$ and achieving maximum connectivity among heterogeneous nodes within the network. Through dynamic reconfiguration, the connectivity reliability of the network exhibits certain characteristics of resistance to internal and external disturbances (such as a potential increase in the number of functional chains), no longer conforming to the strict monotonic decreasing law of classical system reliability. Two basic principles are proposed for dynamic reconfiguration.

#### 4.2.1. Principle of Minimal Movement

Among all feasible reconfiguration schemes, prioritize the one that requires the minimum number of candidate nodes to be repositioned, minimizing energy consumption and reconfiguration latency. When multiple incomplete functional chains coexist in the network, if one functional chain can form a new complete functional chain by calling one candidate node, while another functional chain requires calling at least two candidate nodes to form a new complete functional chain, then the former should be dynamically restructured first according to the principle of minimal movement.

#### 4.2.2. Principle of Minimum-Ordinal Decision Node

For multiple incomplete functional chains that simultaneously satisfy the minimum movement principle, dynamic reconfiguration is prioritized for the incomplete functional chain containing the decision node with the minimum ordinal number, based on whether there is a decision node in the incomplete functional chain and the ordinal number of the decision node. The prerequisite for applying this principle is that the decision node with a smaller ordinal number within the network has higher importance, and the decision node with the largest ordinal number has lower importance. That is, node $N_{1}^{C}$ has the highest importance, while $N_{m_{1}}^{C}$ has the lowest importance.

The paper stipulates that the principle of minimal movement takes precedence over the principle of minimum-ordinal decision node, and the functional chain index value is consistent with the ordinal number of the included decision nodes.

### 4.3. Reconfiguration Execution Steps and Real-Time Calculation Algorithm

Based on the principle of minimal movement and the principle of minimum-ordinal decision node, the paper presents the dynamic reconfiguration steps (DRS) for fixed-formation heterogeneous MANETs:

(1) Check if the failed-functional-chain set is empty. If $l_{F}^{kc}=\emptyset$ (no failed chains require reconfiguration), terminate the reconfiguration process and proceed to step (5); otherwise, proceed to step (2).
(2) Check if the candidate set is empty. If the candidate node set is empty, $V_{Cd}=\emptyset$, proceed to step (5); otherwise, proceed to step (3).
(3) Rank failed functional chains for reconfiguration based on the two principles. Based on the principles of minimum movement and minimum decision nodes, prioritize the dynamic reconfiguration of the failed functional chains in the set $l_{F}^{kc}$.
(4) Reconfigure functional chains in priority order. Traverse all the failed functional chains in the set $l_{F}^{kc}$ according to the priority order determined in step (3). When the reconfiguration of the failed functional chain $l_{j,F}^{kc}$ in the set $l_{F}^{kc}$ is completed, denote it as $l_{j}^{kc}$ and update $l_{F}^{kc}=l_{F}^{kc}\backslash \left\{l_{j,F}^{kc}\right\}$ and $V_{Cd}=V_{Cd}\backslash \left\{N_{i}|N_{i}\in l_{j}^{kc}\right\}$.
(5) Terminate dynamic reconfiguration. This round of dynamic reconfiguration is complete, and the updated sets of failed functional chains $l_{F}^{kc}$ and candidate nodes $V_{Cd}$ for the heterogeneous mobile ad hoc network are output.

A cell array $Cl^{kc}\left(t\right)$ records functional-chain information at time t, with dimension $\max N_{kc}\times \left|V_{type}\right|$. The first dimension $\max N_{kc}$ is the initial functional chain count at t=0, i.e., $\max N_{kc}=N_{kc}\left(0\right)$. The second dimension $\left|V_{type}\right|$ denotes the number of heterogeneous node types that constitute the functional chains. Each cell stores one functional chain and its nodes: $Cl^{kc}\left(0\right)={\left\{l_{1}^{kc},l_{2}^{kc},\ldots ,l_{N_{kc}\left(0\right)}^{kc}\right\}}^{T}$. Based on the above dynamic reconfiguration steps (DRS), the paper designs a real-time algorithm (Algorithm 1) for computing the number of complete functional chains in the heterogeneous MANET.

Input: Binary adjacency matrix $A_{RB}\left(t\right)$ of the heterogeneous functional chain network at time t and functional chain cell array $Cl^{kc}\left(t\right)$ at time t.

Output: Number of complete functional chains $N_{kc}\left(t\right)$ at time t, updated array of functional chain cells $Cl^{kc}\left(t\right)$, and set of failed functional chains $l_{F}^{kc}=\left\{l_{j,F}^{kc}|j=1,2,\ldots \right\}$.

(1) Initialization. Let the number of complete functional chains in the network at the current moment t be denoted by $N_{kc}\left(t\right)=0$, and j=1.
(2) Identify the functional chains and their constituent nodes in the functional chain cell array $Cl^{kc}\left(t\right)$ sequentially. Traverse all functional chains $l_{j}^{kc}$ in the functional chain cell array $Cl^{kc}\left(t\right)$. If the current functional chain has been marked as $l_{j,F}^{kc}$ incomplete, update j=j+1. If $j>\max N_{kc}$, execute step (5). Otherwise, identify the three types of constituent heterogeneous nodes of the current functional chain $l_{j}^{kc}$ and their sequence numbers $\left\{N_{s}^{S},N_{c}^{C},N_{w}^{W}\right\}$, and execute step (3).
(3) Calculate the node index value based on the current complete functional chain composition node sequence number. Using the formula $N_{c}^{C}=N_{c}$, $N_{s}^{S}=N_{s+m_{1}}$, $N_{w}^{W}=N_{w+m_{1}+m_{2}}$, convert the sequence numbers of the three types of nodes into their index values $\left\{N_{s+m_{1}},N_{c},N_{w+m_{1}+m_{2}}\right\}$ in the heterogeneous mobile ad hoc network. Then, proceed to step (4).
(4) Verify the connectivity of the functional chain. If $A_{RB}\left(t\right)\left(s+m_{1},c\right)=A_{RB}\left(t\right)\left(c,w+m_{1}+m_{2}\right)=1$, $a_{RBs+m_{1},c}\left(t\right)=a_{RBc,w+m_{1}+m_{2}}\left(t\right)=1$, then the current complete functional chain $l_{j}^{kc}$ is determined to be in a connected state, and $N_{kc}\left(t\right)=N_{kc}\left(t\right)+1$ is updated. Otherwise, the current functional chain is determined to be in an interrupted state, and the current functional chain $l_{j}^{kc}$ is marked as an element $l_{j,F}^{kc}$ in the set of failed functional chains $l_{F}^{kc}$, and $l_{F}^{kc}=l_{F}^{kc}\cup \left\{l_{j,F}^{kc}\right\}$ is updated. Update j=j+1. If $j>\max N_{kc}$, then proceed to step (5); otherwise, proceed to step (2).
(5) Algorithm ends. Output the number of complete functional chains $N_{kc}\left(t\right)$ at the current moment t, the updated functional chain cell array $Cl^{kc}\left(t\right)$, and the set of failed functional chains $l_{F}^{kc}=\left\{l_{j,F}^{kc}|j=1,2,\ldots \right\}$.

**Algorithm 1.** Real-time algorithm for computing the number of complete functional chains.**Input:**$A_{RB}\left(t\right),$ 
$Cl^{kc}\left(t\right)$
**Output:**$N_{kc}\left(t\right),$ updated $Cl^{kc}\left(t\right),$ $l_{F}^{kc}$1.$\mathrm{Initialize}\;N_{kc}\left(t\right)=0,$ and j=12.**For** $l_{j}^{kc}\in Cl^{kc}\left(t\right)$3.  **If** $l_{j}^{kc}$ is incomplete4.    $l_{j}^{kc}\leftarrow l_{j,F}^{kc},$ 
$l_{F}^{kc}=l_{F}^{kc}\cup \left\{l_{j,F}^{kc}\right\},$ 
j=j+1
5.$//\;\mathrm{Mark}\;l_{j}^{kc}$ 
$\mathrm{as}\;l_{j,F}^{kc}$, and traverse the next 
$l_{j}^{kc}$
6.    **If** $j>\max N_{kc}$7.// Determine whether the algorithm termination condition has been triggered8.      Proceed to step (30)9.    **Else**
10.      Proceed to step (13)11.    **End if**
12.  **Else**
13.    Identify $\left\{N_{s}^{S},N_{c}^{C},N_{w}^{W}\right\}$ in $l_{j}^{kc}$14.  **End if**
15.**End for**16.$N_{c}^{C}=N_{c},$ 
$N_{s}^{S}=N_{s+m_{1}},$ 
$N_{w}^{W}=N_{w+m_{1}+m_{2}}$
17.// Calculate the index value of nodes in $l_{j}^{kc}$18.**If** $A_{RB}\left(t\right)\left(s+m_{1},c\right)=A_{RB}\left(t\right)\left(c,w+m_{1}+m_{2}\right)=1$19.// Verify the connectivity of the functional chain20.  $N_{kc}\left(t\right)=N_{kc}\left(t\right)+1,$ 
j=j+1
21.**Else**22.  $l_{j}^{kc}\leftarrow l_{j,F}^{kc},$ 
$l_{F}^{kc}=l_{F}^{kc}\cup \left\{l_{j,F}^{kc}\right\},$ 
j=j+1
23.**End if**24.**If** $j>\max N_{kc}$25.// Determine whether the algorithm termination condition has been triggered26.  Proceed to step (30)27.**Else**28.  Proceed to step (3)29.**End if**30.Output the result

Complexity Analysis of Algorithm 1.

Let $m=\left|V\right|$ be the total number of nodes, $KC=\max N_{kc}$ be the maximum number of functional chains, and $M=\left|V_{Cd}\right|$ be the candidate node pool size. The algorithm executes at each simulation time step.

(1) Failed-chain detection and candidate-pool construction: For each of the KC functional chains, three node states and two adjacency entries are checked in $O\left(1\right)$ time. The candidate pool $V_{Cd}$ is populated by iterating over nodes within failed chains. In the worst case, all chains fail, yielding $\left|l_{F}^{kc}\right|=KC$ and $\left|V_{Cd}\right|\leq m$. Total: $O\left(KC+m\right)=O\left(m\right)$.
(2) Priority-metric computation and sorting: For each failed chain in $l_{F}^{kc}$, the missing-node count is computed in $O\left(1\right)$ via simple integer arithmetic. Sorting $l_{F}^{kc}$ by primary key (missing count) and secondary key (decision-node index) using a stable comparison sort requires $O\left(\left|l_{F}^{kc}\right|\log\left|l_{F}^{kc}\right|\right)=O\left(m\log m\right)$ in the worst case.
(3) Per-chain reconfiguration: For each $l_{j}^{kc}$ in sorted $l_{F}^{kc}$, identify missing roles ($O\left(1\right)$), then search the candidate pool $V_{Cd}$ for type-matching nodes ($O\left(\left|V_{Cd}\right|\right)$ to filter by type) and verify adjacency connectivity ($O\left(\left|V_{Cd}\right|\right)$ adjacency lookups, each $O\left(1\right)$). The replacement node is selected by taking the minimum index ($O\left(\left|V_{Cd}\right|\right)$).

In the worst case, $\left|l_{F}^{kc}\right|=KC=O\left(m\right)$ and $\left|V_{Cd}\right|=O\left(m\right)$, making this step O($m^{2}$) per time step. However, in practice, $\left|l_{F}^{kc}\right|\ll KC$ and $\left|V_{Cd}\right|\ll m$ because (a) failures accumulate progressively, and (b) the candidate pool is consumed by successful reconfigurations.

Overall per-timestep complexity: Worst case: O($m^{2}$), dominated by the per-chain candidate search. Average case: $O\left(m\times d_{bar}\right)$, where $d_{bar}$ is the average number of candidate nodes examined per failed chain, expected to be significantly smaller than m due to spatial proximity and role-matching constraints.

## 5. Algorithms for Evaluating and Simulating Connectivity Reliability in Heterogeneous MANETs

For clear and precise presentation of the proposed connectivity reliability simulation algorithm, key mathematical notations are summarized in Table 4.

### 5.1. Evaluation Metrics

#### 5.1.1. Number of Functional Chains and Normalized Connectivity Reliability

For a fixed-formation heterogeneous MANET denoted as $G_{fd}$, the maximum number of functional chains $N_{kc}$ corresponds to its fully deployed initial state, i.e.,$\max N_{kc}=N_{kc}\left(0\right)$.

To facilitate comparison of connectivity reliability across different network systems, the evaluation metric is normalized as follows:

(20) $$R_{kc}\left(t\right)=\frac{N_{kc}\left(t\right)}{\max N_{kc}}$$

In this formulation, $R_{kc}\left(t\right)$ denotes the network connectivity reliability computed using the number of intact functional chains at time t, with a range of $\left[0,1\right]$. $R_{kc}\left(t\right)=0$ implies total disconnection of all functional chains and loss of connectivity among all three node types (sensor, decision, and action-execution nodes). $R_{kc}\left(t\right)=1$ indicates normal operation of all functional chains and persistent connectivity among nodes.

Let $N_{kc}^{i}\left(t\right)$ be the number of intact functional chains at time $t\in \left[0,t_{mi}\right]$ in the i-th simulation run. After $N_{simu}$ independent runs, the normalized connectivity reliability is computed as

(21) $$R_{kc}\left(t\right)=\frac{1}{N_{simu}}\sum_{i=1}^{N_{simu}}\frac{N_{kc}^{i}\left(t\right)}{\max N_{kc}}$$

Here, $t_{mi}$ denotes the MANET mission duration, corresponding to the runtime of a single simulation.

#### 5.1.2. Network Quality

Based on the definition of transmission quality in communication networks [15,39], network quality (NQ) is introduced as an evaluation metric. It is defined as the integral of the reliability curve over time, given by

(22) $$NQ=\int_{0}^{+\infty }R_{kc}\left(t\right)dt$$

The connectivity quality of heterogeneous MANETs is derived by integrating the normalized connectivity reliability $R_{kc}\left(t\right)$ over time.

#### 5.1.3. K% Connectivity Reliability

When the mission baseline requires at least K% ($K\in \left(0,100\right)$) of functional chains to remain operational over the mission duration, the K% connectivity reliability metric is defined. The calculation formula is

(23) $$R_{kc,K\%}\left(t\right)=\mathrm{Pr}\left(\frac{N_{kc}\left(t\right)}{\max N_{kc}}\geq K\%\right),\;0<K<100$$

For each i-th simulation experiment, the K% connectivity reliability $R_{kc,K\%}\left(t\right)$ requires judging whether the ratio of remaining functional chains to the maximum chain count meets or exceeds the threshold K% at time t. Accordingly, an indicator function $I_{kc}^{K\%}\left(t\right)$ is introduced:

(24) $$I_{kc,K\%}^{i}\left(t\right)=\left\{\begin{array}{l} 1,\;\frac{N_{kc}^{i}\left(t\right)}{\max N_{kc}}\geq K\%\\ 0,\;\frac{N_{kc}^{i}\left(t\right)}{\max N_{kc}}<K\% \end{array}\right.$$

In the i-th simulation experiment, $I_{kc}^{K\%}\left(t\right)=1$ if $N_{kc}^{i}\left(t\right)\geq \max N_{kc}\times K\%$ (predefined threshold), and 0 otherwise.

After $N_{simu}$ simulation runs, the K% connectivity reliability $R_{kc,K\%}\left(t\right)$ of MANETs at time t is computed as

(25) $$R_{kc,K\%}\left(t\right)=\frac{1}{N_{simu}}\sum_{i=1}^{N_{simu}}I_{kc,K\%}^{i}\left(t\right)$$

### 5.2. Simulation Algorithm for Connectivity Reliability Evaluation

A simulation algorithm for computing the connectivity reliability of fixed-formation heterogeneous MANETs with dynamic reconfiguration is proposed (Algorithm 2). The procedure for each simulation run is detailed below:

**Input:** Graph-theoretic model of heterogeneous MANETs $G=\left(V,E\right)$; mission duration $t_{mi}$.

**Output:** Number of functional chains $N_{kc}\left(t\right)$; indicator function $I_{kc}^{K\%}\left(t\right)$.

(1) Initialize the graph-theoretic model of the heterogeneous MANET. Define node sets: operational nodes $V_{1}=V$, hardware/software failure nodes $V_{2}=\emptyset$, intentional attack failure nodes $V_{3}=\emptyset$, isolation failure nodes $V_{4}=\emptyset$, failed functional chain set $l_{F}^{kc}=\emptyset$, and candidate nodes set $V_{Cd}=\emptyset$.
(2) Identify hardware/software failures. For all nodes $N_{i}\in V_{1}$, compute the failure probability $\mathrm{Pr}\left(S_{N_{i}}\left(t\right)=2\right)$ and compare it with a uniform random number $r\in \left[0,1\right]$. If $\mathrm{Pr}\left(S_{N_{i}}\left(t\right)=2\right)\geq r$, mark $N_{i}$ as failed and its associated chain $l_{j}^{kc}$ as failed. Update $V_{2}=V_{2}\cup \left\{N_{i}\right\}$, $V_{1}=V_{1}\backslash \left\{N_{i}\right\}$, $l_{j}^{kc}=l_{j,F}^{kc}$, $l_{F}^{kc}=l_{F}^{kc}\cup \left\{l_{j,F}^{kc}\right\}$. After traversing all normal nodes in the current heterogeneous MANET, if the condition $V_{1}\neq \emptyset$ is met, proceed to step (3); otherwise, proceed to step (11).
(3) Identify intentional attack failures. For all nodes $N_{i}\in V_{1}$, compute the failure probability $\mathrm{Pr}\left(S_{N_{i}}\left(t\right)=3\right)$ and compare it with a uniform random number $r\in \left[0,1\right]$. If $\mathrm{Pr}\left(S_{N_{i}}\left(t\right)=3\right)\geq r$, mark $N_{i}$ as failed and its functional chain $l_{j}^{kc}$ as failed. Update $V_{3}=V_{3}\cup \left\{N_{i}\right\}$, $V_{1}=V_{1}\backslash \left\{N_{i}\right\}$, $l_{j}^{kc}=l_{j,F}^{kc}$, $l_{F}^{kc}=l_{F}^{kc}\cup \left\{l_{j,F}^{kc}\right\}$. After traversing all normal nodes in the current heterogeneous MANET, if $V_{1}\neq \emptyset$, then proceed to step (4); otherwise, proceed to step (11).
(4) Construct the binary adjacency matrix. For all nodes in the current set $V_{1}$, compute the Euclidean distance matrix using $d_{ij}\left(t\right)=\sqrt{{\left|x_{i}\left(t\right)-x_{j}\left(t\right)\right|}^{2}+{\left|y_{i}\left(t\right)-y_{j}\left(t\right)\right|}^{2}}$. Substitute the distances into the FS-TRG model to obtain $A_{RB}\left(t\right)$ of network at time t.
(5) Identify isolation failures. Using $A_{RB}\left(t\right)$, check all nodes. If node $N_{i}\in V_{1}$ satisfies $\sum_{j\in V_{1}}a_{RBij}=\sum_{j\in V_{1}}a_{RBji}=0$, mark $N_{i}$ as an isolation failure and its chain $l_{j}^{kc}$ as failed. Update $V_{4}=V_{4}\cup \left\{N_{i}\right\}$, $V_{1}=V_{1}\backslash \left\{N_{i}\right\}$, $l_{j}^{kc}=l_{j,F}^{kc}$, $l_{F}^{kc}=l_{F}^{kc}\cup \left\{l_{j,F}^{kc}\right\}$. If $V_{1}\neq \emptyset$ are met, proceed to step (6); otherwise, proceed to step (11).
(6) Update the candidate node set for MANET reconfiguration. Traverse all incomplete functional chains $\left\{l_{F,j}^{kc}|j=1,2,\ldots \right\}$ in the set of failed functional chains $l_{F}^{kc}$, and if there are constituent nodes $N_{i}$ that are in normal working condition, update $V_{Cd}=V_{Cd}\cup \left\{N_{i}\right\}=\left\{N_{i}|N_{i}\in l_{j,F}^{kc},\;S_{N_{i}}=1,\;i=1,2,\ldots ,\;j=1,2,\ldots \right\}$.
(7) Perform dynamic reconfiguration of the heterogeneous MANET. Execute the proposed DRS steps based on the principles of minimal movement and minimum-ordinal decision node.
(8) Compute the current number of intact functional chains. Execute Algorithm 1 to output $N_{kc}\left(t\right)$ and update the failed functional chain set $l_{F}^{kc}=\left\{l_{j,F}^{kc}|j=1,2,\ldots \right\}$.
(9) Update node positions. For all nodes in the current set $V_{1}$, calculate and update the direction vectors $O_{i}\left(t+\Delta t\right)$, coordinates, and other information for each node at the next moment t+Δt using the improved Couzin-leader model.
(10) Update the simulation clock. t=t+Δt. If $t\leq t_{mi}$, proceed to step (2) of the algorithm and continue the iterative operation; otherwise, execute step (11) of the algorithm.
(11) End of algorithm. Output the data from this simulation experiment: the number of complete functional chains $N_{kc}\left(t\right)$ and the indicator function $I_{kc}^{K\%}\left(t\right)$.

**Algorithm 2.** Simulation algorithm for computing the connectivity reliability of heterogeneous MANETs with dynamic reconfiguration.**Input:**$G=\left(V,E\right),$ 
$t_{mi}$
**Output:**$N_{kc}\left(t\right),$ 
$I_{kc}^{K\%}\left(t\right)$
1.$\mathrm{Initialize}\;G=\left(V,E\right)$2.// Determine the number of nodes $m=\left|V\right|$ and the index of each node $N_{i}\left(i=1,2,\ldots ,m\right)$
3.$\mathrm{Set}\;V_{1}=V,$ $V_{2}=\emptyset ,$ $V_{3}=\emptyset ,$ $V_{4}=\emptyset ,$ $l_{F}^{kc}=\emptyset ,$ and $V_{Cd}=\emptyset .$4.// Corresponding to the 6 sets5.**For** 
$N_{i}\in V_{1},$ 
$\mathrm{calculate}\;\mathrm{Pr}\left(S_{N_{i}}\left(t\right)=2\right)$
6.  **If** 
$\mathrm{Pr}\left(S_{N_{i}}\left(t\right)=2\right)\geq r,$ 
$r\in \left[0,1\right]$
7.    $\;\mathrm{Update}\;V_{2}=V_{2}\cup \left\{N_{i}\right\},$ 
$V_{1}=V_{1}\backslash \left\{N_{i}\right\},$ 
$l_{j}^{kc}\leftarrow l_{j,F}^{kc},$ 
$l_{F}^{kc}=l_{F}^{kc}\cup \left\{l_{j,F}^{kc}\right\}$
8.  **End if**
9.**End for**10.// Determine whether a node has experienced a hardware/software failure11.**For** 
$N_{i}\in V_{1},$ 
$\mathrm{calculate}\;\mathrm{Pr}\left(S_{N_{i}}\left(t\right)=3\right)$
12.  **If** 
$\mathrm{Pr}\left(S_{N_{i}}\left(t\right)=3\right)\geq r,$ 
$r\in \left[0,1\right]$
13.    $\;\mathrm{Update}\;V_{3}=V_{3}\cup \left\{N_{i}\right\},$ 
$V_{1}=V_{1}\backslash \left\{N_{i}\right\},$ 
$l_{j}^{kc}\leftarrow l_{j,F}^{kc},$ 
$l_{F}^{kc}=l_{F}^{kc}\cup \left\{l_{j,F}^{kc}\right\}$
14.  **End if**
15.**End for**16.// Determine whether a node has experienced an intentional attack failure17.$\mathrm{Generate}\;A_{RB}\left(t\right)$18.**For** 
$N_{i}\in V_{1}$
19.  **If** 
$\sum_{j\in V_{1}}a_{RBij}=\sum_{j\in V_{1}}a_{RBji}=0,$ 
j≠i
20.    $\;\mathrm{Update}\;V_{4}=V_{4}\cup \left\{N_{i}\right\},$ 
$V_{1}=V_{1}\backslash \left\{N_{i}\right\},$ 
$l_{j}^{kc}\leftarrow l_{j,F}^{kc},$ 
$l_{F}^{kc}=l_{F}^{kc}\cup \left\{l_{j,F}^{kc}\right\}$
21.  **End if**
22.**End for**23.// Determine whether a node has experienced isolation failure24.Update $V_{Cd}$25.Perform DRS26.// Execute the proposed dynamic reconfiguration steps27.Run Algorithm 128.// Compute the current number of intact functional chains29.Output $N_{kc}\left(t\right)$ and update $l_{F}^{kc}$30.Run the improved Couzin-leader model31.// Update node positions32.**If** 
$V_{1}\neq \emptyset$
33.  t=t+Δt
34.    **If** 
$t<t_{mi}$
35.      Proceed to step (5)36.    **Else**
37.      Proceed to step (42)38.    **End if**
39.**Else**40.  Proceed to step (42)41.**End if**42.Output the result

Complexity Analysis of Algorithm 2.

Let $m=\left|V\right|$ be the total number of nodes, n the number of edges, T the number of simulation time steps ($T=t_{mi}/\Delta t$), and $N_{simu}$ the number of Monte Carlo trials. The per-timestep cost decomposes as follows:

(1) Movement: $O\left(m\times d_{avg}\right)$ where $d_{avg}$ is the average degree within communication range. In the worst dense case, $d_{avg}=O\left(m\right)$, giving $O\left(m^{2}\right)$. In practical MANET scenarios, $d_{avg}\ll m$ due to bounded communication radii.
(2) Adjacency: $O\left(m^{2}\right)$ due to all-pairs distance computation via pdist2 (MATLAB). This is the dominant term for medium-to-large m.
(3) Fault injection: $O\left(m\right)$ linear scan over all nodes.
(4) Isolation detection: $O\left(m\right)$ linear scan.
(5) Dynamic reconfiguration (Algorithm 1): $O\left(m\right)$ worst case, $O\left(m\times d_{bar}\right)$ average.
(6) Chain counting and graph analysis: $O\left(KC+\left|E\right|\right)=O\left(m+n\right)$.

Overall per-timestep: O($m^{2}$), dominated by adjacency computation.

## 6. Case Study

### 6.1. Heterogeneous Network Model Setup

A simulation case is constructed for a heterogeneous MANET denoted as $G_{DR}$, which comprises 210 nodes sequentially numbered from $N_{1}$ to $N_{210}$. The network consists of 70 decision nodes $N^{C}=\left\{N_{c}^{C}|c=1,2,\ldots ,70\right\}$, 70 sensor nodes $N^{S}=\left\{N_{s}^{S}|s=1,2,\ldots ,70\right\}$, and 70 action execution nodes $N^{W}=\left\{N_{w}^{W}|w=1,2,\ldots ,m_{3}=70\right\}$. The simulation mission duration is set to $t_{mi}=1\;h$, and the time step is Δt=1 s. All simulations run in MATLAB R2023b with 100 independent trials, and reliability metrics are averaged for statistical stability. To ensure the reproducibility of the Monte Carlo simulations, the MATLAB pseudo-random number generator was initialized at the beginning of each independent trial with a fixed seed derived from the trial index (seed = trial index + 1000). This guarantees that each trial uses an independent yet deterministic random sequence, enabling exact replication of all reported results by any researcher using the same MATLAB version and the same seed values. Table 5 summarizes the simulation parameters and their corresponding values.

For the improved Couzin-leader and isolation failure models, the network includes 70 leader nodes (decision nodes $N^{C}=\left\{N_{i}|i=1,2,\ldots ,70\right\}$), where $N_{1}$ is the top-level control node. The other 140 nodes ($N^{S}\cup N^{W}=\left\{N_{i}|i=71,72,\ldots ,210\right\}$) act as followers. At t=0, the sensor node $N_{j}^{S}$ and action execution node $N_{j}^{W}$ of the same functional chain $l_{j}^{kc}$ (j=1,2,…,70) are deployed in a circle centered at $N_{j}^{C}$ with radius $2d_{Co}$, ensuring 70 intact functional chains.

### 6.2. Analysis of Simulation Experiment Results

**Figure 5:**
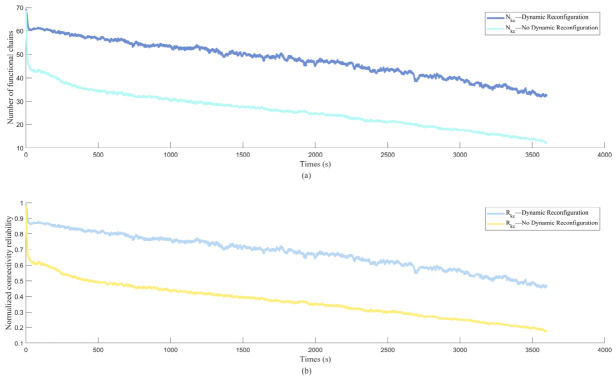
Comparison of functional chain quantity and normalized reliability. (**a**) Comparison of functional chain quantity; (**b**) comparison of normalized connectivity reliability.

To investigate the effect of dynamic reconfiguration on connectivity reliability, a control network $G_{NDR}$ without reconfiguration is built with identical parameters. Figure 5 plots the functional chain count $N_{kc}\left(t\right)$ and normalized connectivity reliability $R_{kc}\left(t\right)$ from the simulations. The two metrics show identical trends, as $R_{kc}\left(t\right)$ is the normalized form of $N_{kc}\left(t\right)$.

As shown in Figure 5a, the functional chain count drops sharply in the initial stage $t\in \left(0,64\right]$. The heterogeneous network $G_{DR}$ considering dynamic reconfiguration decreases to $N_{kc}\left(t\right)=60.16\pm 0.447$ at time t=35, while the heterogeneous network $G_{NDR}$ without dynamic reconfiguration decreases to $N_{kc}\left(t\right)=42.32\pm 0.703$ at time t=64. This steep decline arises from the quick decoupling of initially compact functional chains under high node mobility. At the initial time (t=0) of the network, 70 complete functional chains ($N_{kc}\left(0\right)=70$) are set through node allocation and grouping. In each functional chain, both the sensor node and action execution node maintain a close distance ($d_{Th}$) to the decision node, so the reliability of all links is 1. After the simulation experiment begins, at time t≤64, the probability of node hardware/software failure and intentional attack is extremely low, and node failure is not the main reason for the early decrease in the number of complete functional chains. Instead, nodes move at 20~60 km/h, traveling 5.6~16.7 m per time step Δt. Driven by the improved Couzin-leader model, initially clustered nodes spread out due to repulsion and task-directed motion. After only 2~6 simulation time steps, nodes may move beyond 35 m ($\alpha d_{Th}$), at which point the reliability of links begins to decrease. When the movement exceeds 70 m ($d_{Th}$), links between nodes break. In addition, there is also inter-chain attraction and alignment between nodes of different functional chains, which may pull nodes away from their original functional chain structure. Therefore, at the initial stage ($t\in \left(0,64\right]$) of the simulation experiment, the Euclidean distance between a large number of nodes within functional chains quickly exceeds the reliable communication range, leading to link breakage and a sharp decrease in the number of functional chains.

Without dynamic reconfiguration, a broken chain remains permanently failed, as spontaneous re-clustering of the three nodes is highly unlikely. However, the probability of this occurring is low without an active reconfiguration mechanism. Therefore, the number of functional chains in the control group network model $G_{NDR}$ decreases the fastest (from 70 to 42.32±0.703). With the proposed reconfiguration (following minimal movement and minimum-ordinal decision node principles), the algorithm quickly selects operable candidate nodes to replace faulty ones and rebuild complete chains. The decline in the number of complete functional chains is partially “recovered” (from 70 to 60.16±0.447), significantly curbing the initial deterioration of network connectivity reliability. This comparison highlights the application value of dynamic reconfiguration in heterogeneous MANETs, which can effectively maintain the number of functional chains and ensure network performance even during adverse periods of rapid network topology changes.

**Figure 6:**
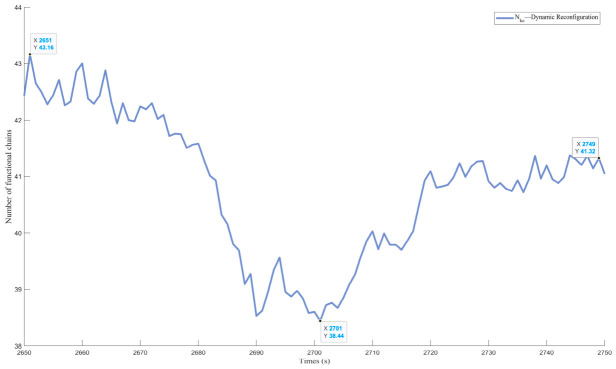
Example of V-shaped fluctuation in the number of dynamically reconfigured network functional chains.

Over $t\in \left[500,3600\right]$, the control network $G_{NDR}$ shows a steady decline, while the reconfigured network $G_{DR}$ exhibits clear V-shaped fluctuations. Taking the time period $t\in \left[2650,2750\right]$ as an example, the number of network $G_{DR}$ functional chains $N_{kc}\left(2651\right)=43.16\pm 1.886$ decreased to $N_{kc}\left(2701\right)=38.44\pm 2.367$, and after dynamic reconfiguration, the number of functional chains increased to $N_{kc}\left(2749\right)=41.32\pm 2.160$, representing a 7.49% increase compared to time t=2701, and has recovered to 95.74% of the time t=2651, as shown in Figure 6.

As listed in Table 6, dynamic reconfiguration yields significant improvements by the end of the simulation. At time t=3600, the number of functional chains in the dynamically reconfigured network $G_{DR}$ was 20.67 more than that in the control group network $G_{NDR}$, and its normalized reliability $R_{kc}\left(3600\right)$ was 0.2952 higher than that of the control group network $G_{NDR}$. By integrating and summing the normalized connectivity reliability $R_{kc}$ over the duration of the simulation experiment, it was ultimately found that the dynamically reconfigured network $G_{DR}$ was 82.9630% higher than the control group network $G_{NDR}$.

**Figure 7:**
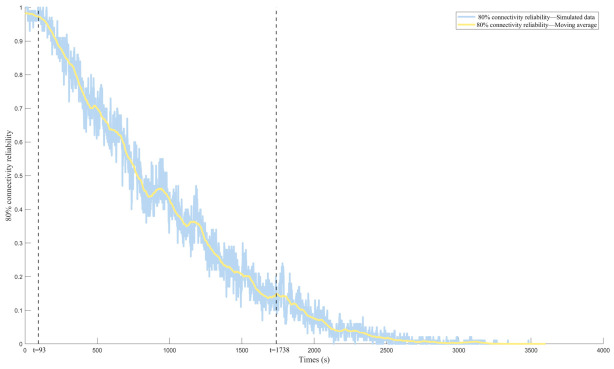
The 80% connectivity reliability and its three-stage segmentation in heterogeneous MANETs.

Figure 7 depicts the time-varying probability that the reconfigured network $G_{DR}$ maintains at least 80% of its functional chains, based on 100 Monte Carlo trials. To analyze 80% connectivity reliability $R_{kc,80\%}\left(t\right)$, the data was filtered using a smooth curve (moving average curve) to remove high-frequency noise and highlight its changing trend. The smooth curve was obtained by calculating a moving average with a sliding window of 100. The dynamically reconfigurable network $G_{DR}$ is last satisfied $\frac{N_{kc}\left(t\right)}{\max N_{kc}}\geq K\%$ at time t=684, at which point the number of functional chains $N_{kc}\left(684\right)=55.91\pm 0.891$ and the 80% connectivity reliability is $R_{kc,80\%}\left(684\right)=0.7987\pm 0.013$. To capture the relative changes in K% connectivity reliability $R_{kc,K\%}\left(t\right)$ fluctuations, an automatic segmentation algorithm based on slope-volatility detection was developed. The algorithm proceeds as follows: (1) the raw $R_{kc,K\%}\left(t\right)$ curve is smoothed using a moving-average filter with a window of 100 time steps; (2) the local slope of the smoothed signal is computed via first-order differencing and is itself smoothed; (3) the moving standard deviation of the smoothed slope, termed slope volatility, is calculated as a stage-discrimination metric; (4) an adaptive threshold is determined as the median of the slope volatility plus 1.5 times its median absolute deviation (MAD); (5) contiguous time intervals during which the slope volatility exceeds this threshold for at least 60 s are identified as the mid-stage fluctuation period; and (6) the onset of the first such interval defines the Stage I-to-II transition time $T_{1}$, and the offset of the last such interval plus half the window width defines the Stage II-to-III transition time $T_{2}$. This automated, data-driven procedure ensures that the stage classification is objective and reproducible, independent of visual inspection or subjective judgment. The dividing points for the three stages are times $T_{1}=93$ and $T_{2}=1738$, respectively corresponding to $R_{kc,80\%}\left(93\right)=0.9722$ and $R_{kc,80\%}\left(1738\right)=0.1482$. Overall, the network exhibits a process of initial stability, mid-term fluctuations, and later stability.

Early stable period (0<t<93): The probability remains close to 1, with occasional minor fluctuations. All functional chains are initially tightly deployed to ensure connectivity and reach a full complement of 70 chains. The probability of node failure is extremely low, and node failure characteristics are not the main factor affecting functional chain failure. Nodes begin to move according to the improved Couzin-leader model, but within the first few tens of seconds, the distance between most nodes within the chain has not exceeded the reliable communication range, and the links have not been largely interrupted. The functional chains remain intact, so the probability remains stable near 1.

The mid-term fluctuation period (93<t<1738): It is characterized by a continuous decline in probability and significant oscillations. As the simulation time progresses, the effect of node movement leading to a large number of link disconnections gradually becomes apparent. The nodes within the chain gradually disperse under the influence of four forces: repulsion, attraction, alignment, and targeting. Many link distances exceed the communication threshold and disconnect, reducing the number of fully functional chains. At the same time, the accumulation of node hardware/software failures and deliberate attacks further reduces the number of available candidate nodes. The dynamic reconfiguration mechanism plays a key role in this stage: whenever a functional chain fails, the reconfiguration algorithm immediately searches for nodes of the same type from candidate nodes for reorganization, partially restoring the number of functional chains. This repeated iteration of “destruction-reconfiguration” causes the number of fully functional chains to repeatedly cross the 80% threshold and the indicator function $I_{kc}^{K\%}\left(t\right)$ to frequently jump, resulting in a sharp oscillation and overall decline in the probability curve. In the latter half of the mid-term, as the globally available candidate node resources gradually dwindle, the success rate of reconfiguration becomes increasingly lower, the overall trend of functional chain numbers declines, and the center of probability oscillation gradually decreases.

Late-stage stable period (1738<t<3600): The probability drops to a lower level, and fluctuations significantly weaken. In the heterogeneous network, a large number of nodes have failed due to hardware/software failures, intentional attacks, or isolation, and the remaining number of nodes that are working normally is no longer sufficient to support a complete functional chain that meets the threshold requirements. Even dynamic reconfiguration cannot compensate for the resource shortage. At this time, the number of functional chains is consistently below the threshold, indicating that the indicator function $I_{kc}^{K\%}\left(t\right)$ takes the value of 0 most of the time; thus, the probability remains stable at a low level. Occasionally, a few functional chains are temporarily restored due to random movement or reconfiguration, but this is not enough to form sustained fluctuations. The 80% connectivity reliability $R_{kc,80\%}\left(t\right)$ curve tends to stabilize, indicating that the heterogeneous network has entered a quasi-steady state: the remaining nodes are in dynamic equilibrium, and the overall connectivity remains at a lower level.

**Figure 8:**
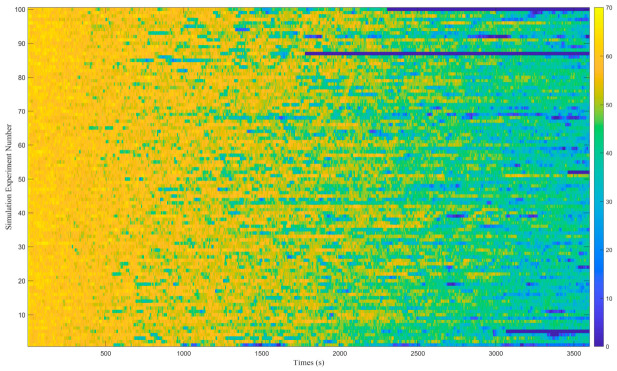
Heatmap of functional chain quantity.

Figure 8 is a heatmap of complete functional chains in the dynamically reconfigured heterogeneous MANET over the full mission duration. The horizontal axis represents simulation time (t = 0 to 4000 s); and the vertical axis represents the 70 functional chains (indexed 1 to 70). Each cell encodes whether chain $l_{j}^{kc}$ is complete (connected S→D→A path) at time t. The color gradient transitions from yellow (chains intact, connectivity healthy) to purple (chains failed). Three temporal regimes are visually discernible: (i) $0\sim T_{1}$: the heatmap is predominantly yellow, with only sporadic dark spots corresponding to the initial stable period where nearly all 70 chains remain connected; (ii) $T_{1}\sim T_{2}$: yellow regions progressively shrink, and purple regions expand—this is the mid-stage fluctuation period, reflecting repeated destruction–reconfiguration cycles. The color field is visibly turbulent, with localized yellow patches reappearing after reconfiguration events; and (iii) $T_{2}\sim t_{mi}$: the heatmap stabilizes in a predominantly purple hue as the candidate node pool is exhausted and reconfiguration can no longer sustain a significant number of complete chains, consistent with the late-stage degradation period identified in the probability curve.

**Figure 9:**
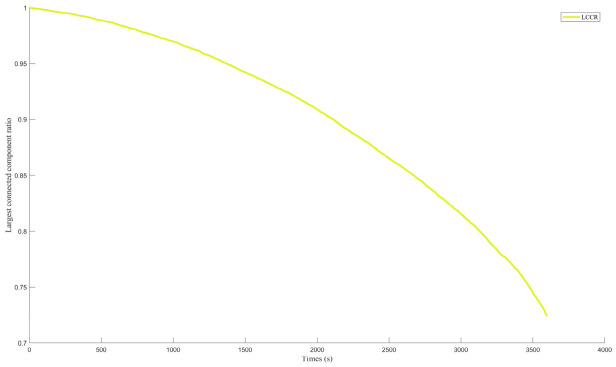
The largest connected component ratio in the network.

A comparison is conducted with a common metric in connectivity analysis: the largest connected component ratio (LCCR). The LCCR at time t is defined as the quotient of the node count in the largest connected component of the network divided by the total number of nodes in the original network. As Figure 9 shows, although the largest connected component ratio $LCCR\left(t\right)$ of the dynamically reconfigured network $G_{DR}$ shows a significant downward trend as the simulation time progresses, the ratio at the end time is 0.7240, which is much higher than the normalized connectivity reliability $R_{kc}\left(3600\right)=0.4681$ and 80% connectivity reliability $R_{kc,80\%}$. This discrepancy reveals that purely topology-based reliability analysis (ignoring node functional heterogeneity and chain coupling) leads to large evaluation errors: the number of available connected nodes in the network at any given time cannot correspond to the number of remaining complete functional chains, and network topology connectivity is not equivalent to normal network functionality. Although the former can effectively support the dynamic reorganization of complete functional chains, only when the available connected nodes simultaneously meet the requirements of node heterogeneity can the number of nodes be proportionally related to the number of functional chains. Otherwise, the network connectivity reliability evaluation results based on the largest connected component ratio LCCR will mislead the operation and maintenance management and task deployment of heterogeneous networks.

To assess the robustness of the conclusions reported above and to further reveal the mechanistic pathways through which key parameters shape connectivity reliability evolution, six groups of parametric sensitivity experiments were conducted. All results are reported as means ± 95% confidence intervals based on 100 independent Monte Carlo trials. Due to space limitations, the experimental data are presented in tabular form (Appendix A).

Experiment 1 (network scale). The total number of nodes $N_{total}$ was varied in {90, 150, 210, 270, 330} while maintaining the S:D:A = 1:1:1 ratio. All other parameters were kept at their baseline values. The deployment area was held constant—reflecting scenarios where the operational space is fixed by task geography and network density varies naturally with the number of deployed nodes.

**Figure 10:**
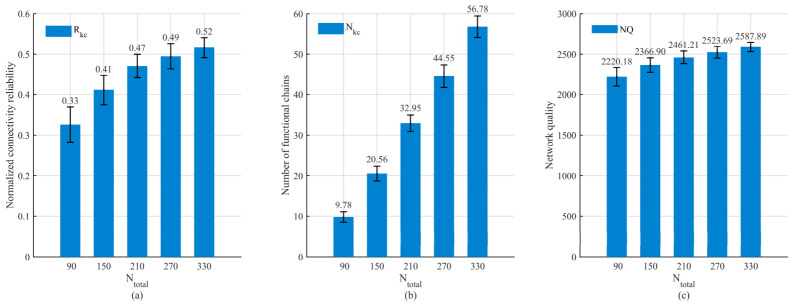
Parametric analysis of network scale. (**a**) Normalized connectivity reliability as the dependent variable; (**b**) Number of functional chains as the dependent variable; (**c**) Network quality as the dependent variable.

As shown in Figure 10, the normalized connectivity reliability $R_{kc}$ increases monotonically with $N_{total}$ but exhibits diminishing marginal returns: the increase from $N_{total}=210$ to $N_{total}=270$ (a 28.6% increase in node count) yields only a 5.1% improvement in $R_{kc}$. This result corroborates the candidate–node–pool saturation hypothesis—beyond a critical network density, newly added nodes are constrained not by the absolute size of the candidate pool but by spatial proximity requirements and role-matching constraints. The confidence intervals narrow progressively with increasing $N_{total}$, indicating that larger networks produce more stable Monte Carlo estimates.

Experiment 2 (maximum velocity). The maximum node velocity $v_{\max}$ was varied in {11.11, 13.89, 16.67, 19.44, 22.22} m/s (corresponding to 40, 50, 60, 70, 80 km/h) while keeping the minimum velocity $v_{\min}=5.56$ m/s (20 km/h) and all other Couzin-leader model parameters at baseline values.

**Figure 11:**
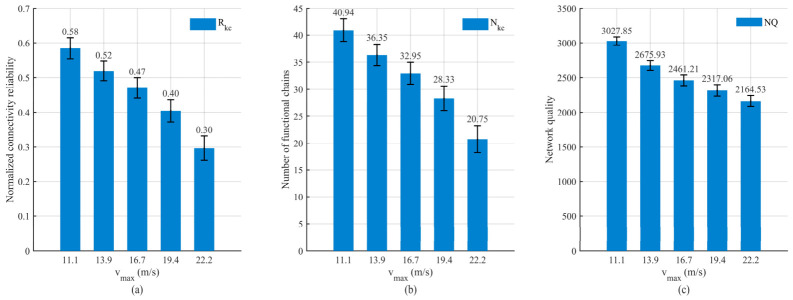
Parametric analysis of maximum node velocity. (**a**) Normalized connectivity reliability as the dependent variable; (**b**) Number of functional chains as the dependent variable; (**c**) Network quality as the dependent variable.

As shown in Figure 11, the normalized connectivity reliability $R_{kc}$ decreases monotonically with increasing $v_{\max}$ over the tested range. The negative effect of link instability induced by higher mobility dominates the positive effect of accelerated candidate node discovery. The monotonic trend does not preclude the existence of an optimal velocity below 11.11 m/s—it leaves open the possibility of a peak at lower speeds.

Experiment 3 (failure rate). The hardware failure rate λ was scaled by {0.1, 0.5, 1.0, 2.0, 5.0} times the baseline value $\lambda_{0}$. The intentional attack failure rate was scaled proportionally. All other parameters were kept at baseline values.

**Figure 12:**
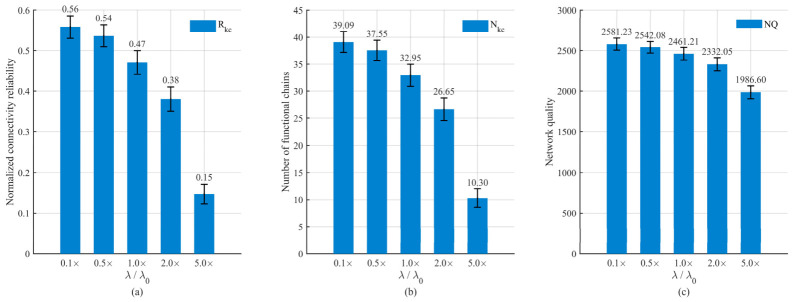
Parametric analysis of failure rate. (**a**) Normalized connectivity reliability as the dependent variable; (**b**) Number of functional chains as the dependent variable; (**c**) Network quality as the dependent variable.

The failure rate λ exhibits the strongest relative impact among the continuous parameters (max/min ratio = 3.80×), confirming it as a critical control variable (Figure 12). At 5.0× the baseline failure rate, only 10.30 ± 1.70 complete functional chains survive at the terminal time (an 85.3% degradation relative to the baseline configuration). This indicates the existence of a critical failure-rate threshold between 2.0× and 5.0×, beyond which the dynamic reconfiguration scheme (DRS) can no longer sustain meaningful functional chain connectivity.

Experiment 4 (communication distance threshold). The communication distance threshold $d_{Th}$ was scaled by {0.6, 0.8, 1.0, 1.2, 1.5} times the baseline value of 70 m. All other parameters were kept at baseline values.

**Figure 13:**
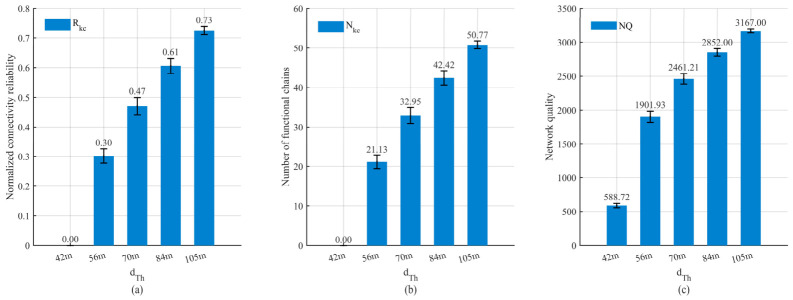
Parametric analysis of communication distance threshold. (**a**) Normalized connectivity reliability as the dependent variable; (**b**) Number of functional chains as the dependent variable; (**c**) Network quality as the dependent variable.

The communication distance threshold $d_{Th}$ produces the largest absolute impact among all tested parameters (Figure 13). At 0.6× baseline (42 m), all nodes become mutually isolated, and the network experiences complete functional collapse ($R_{kc}=0$, $N_{kc}=0$). This result confirms that link reliability is a necessary condition for functional chain connectivity. Even when the communication range is elevated to 1.5× baseline, the three-stage temporal pattern persists (nodes continue to be depleted), thereby corroborating the asymmetric link–node coupling: improved communication capability alone cannot compensate for the exhaustion of the candidate node pool.

Experiment 5 (reconfiguration strategy ablation). Five strategy variants were compared: S0—no dynamic reconfiguration (NoReconf), S1—random reconfiguration (Random), S2—greedy reconfiguration selecting the nearest candidate (Greedy), S3—reconfiguration based solely on the Principle of Minimal Movement (MinMove), and S4—DRS incorporating both the Principle of Minimal Movement and the Principle of Minimum-Ordinal Decision Node. All other parameters were kept at baseline values.

**Figure 14:**
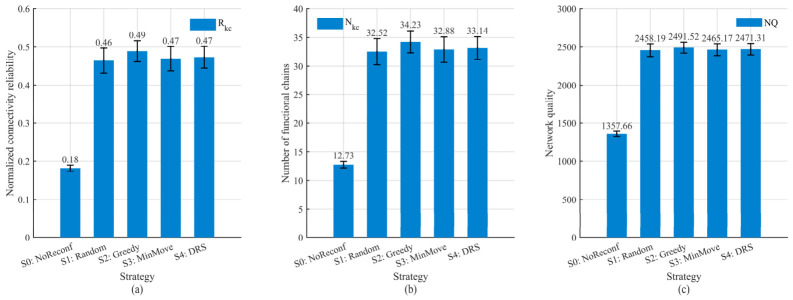
Reconfiguration strategy ablation. (**a**) Normalized connectivity reliability as the dependent variable; (**b**) Number of functional chains as the dependent variable; (**c**) Network quality as the dependent variable.

The ablation analysis (Figure 14) decomposes the total DRS advantage over NoReconf in terms of normalized connectivity reliability $R_{kc}$ and network quality NQ as follows: (S1–S0) captures the baseline reconfiguration value contributed by random reconfiguration; (S2–S1) captures the spatial efficiency gain from selecting the nearest candidate node; (S3–S2) indicates that under the present experimental parameterization, the MinMove strategy alone slightly underperforms the Greedy strategy; and (S4–S3) shows a minimal contribution from the Principle of Minimum-Ordinal Decision Node. Notably, all active strategies (S1 through S4) cluster within a narrow band of $R_{kc}$ = 0.465 ± 0.032 to 0.489 ± 0.028—well above the NoReconf baseline—indicating that any form of structured dynamic reconfiguration dramatically outperforms no reconfiguration, while the marginal differences among strategies are modest. The Greedy strategy (S2) achieves the highest terminal $R_{kc}$ (0.489), suggesting that spatial efficiency may outweigh conflict resolution when the candidate pool is well provisioned.

Experiment 6 (reconfiguration period). The reconfiguration execution period $T_{RP}$ was varied in {1, 5, 10, 15, 20} s. All scenarios employ the DRS strategy; all other parameters were kept at baseline values.

**Figure 15:**
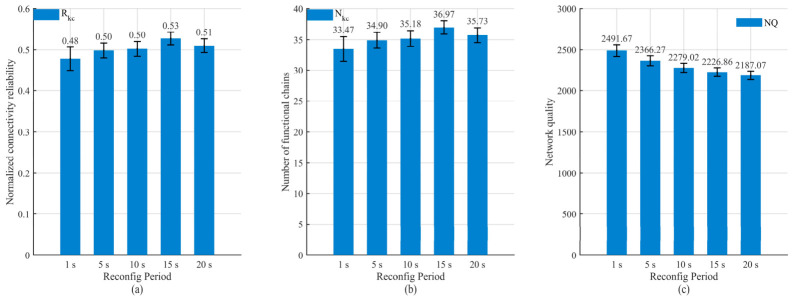
Parametric analysis of reconfiguration period. (**a**) Normalized connectivity reliability as the dependent variable; (**b**) Number of functional chains as the dependent variable; (**c**) Network quality as the dependent variable.

As shown in Figure 15, a counterintuitive result emerges: the terminal normalized connectivity reliability $R_{kc}$ improves as the reconfiguration period lengthens (from 0.478 ± 0.029 at $T_{RP}=1\;s$ to 0.528 ± 0.015 at $T_{RP}=15\;s$), whereas the network quality NQ degrades monotonically (from 2491.7 ± 73.2 to 2187.1 ± 51.3). This asymmetry arises because less frequent reconfiguration leaves more nodes in the uncommitted candidate pool—inflating the terminal snapshot—while simultaneously allowing greater cumulative degradation between reconfiguration windows. The result underscores the importance of reporting both the integral metric NQ (capturing the full temporal experience) and the terminal metric $R_{kc}$ (capturing the endpoint state). From an operational perspective, $T_{RP}=5\;s$ offers a near-optimal compromise: $R_{kc}$ improves by 4.3% over real-time reconfiguration, while NQ degrades only modestly by 5.0%.

Using the baseline configuration data, the K% connectivity reliability threshold was post-processed across K in {60, 70, 80, 90, 95}, and the slope-fluctuation automatic segmentation algorithm was applied independently to each K value.

**Figure 16:**
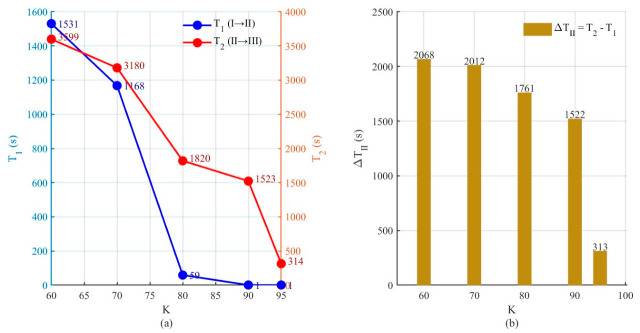
Effect of K-value variation on the three-stage model. (**a**) Stage I to II transition time and Stage II to III transition time; (**b**) The duration of Stage II $\Delta T_{\mathrm{II}}$.

As shown in Figure 16, the three-stage temporal pattern is confirmed across all tested K values. However, the stage boundaries exhibit strong K-dependence: the first-stage duration $T_{1}$ collapses from 1531 s at K=60 to merely 1 s at K≥90, indicating that under demanding thresholds, the network experiences virtually no initial stable period. The second-stage duration $\Delta T_{\mathrm{II}}$ decreases monotonically from 2068 s at K=60 to 313 s at K=95. This analysis demonstrates that the three-stage classification is not an artifact of the K=80 choice but a structural property of the system; however, the specific boundary values are K-dependent and must be interpreted relative to the operational threshold of interest.

## 7. Conclusions

The paper focuses on the dynamic reconfiguration mechanism present in heterogeneous MANETs, examining its impact on the specified functionality of heterogeneous networks within a predetermined timeframe. It proposes a connectivity reliability evaluation metric for quantification and verifies the effectiveness of the reliability analysis through simulation experiments. Combining theoretical analysis with simulation results, the paper draws the following conclusions:

The connectivity of high-dynamic MANETs inherently exhibits volatility. Setting the initial state as “fully connected” is an idealized starting point, and the network will rapidly evolve into a natural topology that is more in line with mobility rules and communication constraints during subsequent movement. This early rapid change is part of the network self-organization process, similar to the approach of physical systems from a non-equilibrium initial state to an equilibrium state. At the same time, networks with dynamic reconfiguration can partially withstand this impact, which precisely demonstrates the effectiveness of dynamic reconfiguration.

Through analyzing 80% connectivity reliability $R_{kc,80\%}\left(t\right)$, it is found that the dynamic reconfiguration mechanism primarily operates in the middle stage of the network. At this time, both the number of failed functional chains and the number of candidate nodes are continuously increasing, providing prerequisites for dynamic reconfiguration. In the early stage of the task phase, the number of failed functional chains is relatively small, and in the later stage of the task phase, there are fewer remaining normal working nodes in the network, both of which affect the effectiveness of the dynamic reconfiguration mechanism. The characteristics of these three stages indicate that dynamic reconfiguration effectively buffers the deterioration of connectivity reliability in the middle stage, but it cannot prevent the network from ultimately degrading due to resource depletion.

At the end of the simulation experiment, the dynamic reconfiguration mechanism had a significant positive impact on the number of remaining complete functional chains, normalized connectivity reliability, and network quality in heterogeneous MANETs. This impact can effectively support the network system to function and continue executing its intended tasks.

Several limitations of the present study should be acknowledged. The conclusions are simulation-driven and have not been validated against field data from deployed heterogeneous MANETs. The current model assumes intra-class node capability homogeneity and evaluates structural connectivity rather than functional chain performance; two connected chains of different quality are treated equivalently. The reconfiguration process itself is modeled as instantaneous, without accounting for its communication overhead or energy cost. Building on these limitations, future work will extend the functional chain model to *n*-stage architectures with relaxed pairing constraints, incorporate intra-class capability heterogeneity into reconfiguration decisions, develop performance-based functional chain metrics, and introduce overhead models to analyze the cost–benefit trade-off of dynamic reconfiguration—ultimately providing decision support for heterogeneous MANET management through connectivity reliability analysis that accounts for both structural integrity and functional performance.

## Figures and Tables

**Table 1:** Evaluation object: communication links vs. functional chains.

Dimension	Existing Metrics	Functional Chain Connectivity Reliability (This Paper)
Assessment object	Communication links/paths: whether an edge is operational, whether an end-to-end path exists. The core question is “Can two points communicate?”	Functional chains: whether a Sensing→Decision→Action closed loop is structurally complete. The core question is “Does a functional chain possess all required role-specific nodes in a connected configuration?”
Failure criterion	Failure of any edge or node reduces topological connectivity. Assesses “structural integrity of the network graph.”	Failure of any single role-specific node in a chain renders the entire chain non-functional. Assesses “completeness of the functional closed loop.”
Recovery mechanism	Rerouting: traffic is redirected through alternative paths. Node functional roles are irrelevant.	Dynamic reconfiguration: a failed node is replaced by a candidate node of the same functional role, re-closing the chain. Node functional roles are the binding constraints.
Core question	How robust is the network itself?	Can the network sustain its mission functionality through active management?

**Table 2:** Evaluation granularity: network-level vs. mission-level.

Dimension	Network-Level Granularity	Mission-Level Granularity (This Paper)
Measurement approach	Network-wide aggregated performance: largest connected component ratio, K-terminal reliability, all-terminal reliability—the network is treated as an undifferentiated whole.	Per-chain functional tracking: the status of each individual functional chain is independently monitored—is it intact? What candidate reconfiguration resources are available? This yields a fine-grained mission-level reliability profile.
Cross-scale comparison	Relies on normalization but does not distinguish functional differences. Networks of different scales may yield similar metric values despite vastly different operational capabilities.	The number of functional chains directly reflects mission scale. $R_{kc}\left(t\right)$ (Section 5.1) enables functionally meaningful cross-scale normalization—networks with different node counts can be compared on an equal functional footing.
Information loss	Relatively high. Simulations (Section 6.2) reveal that at the termination point, the largest connected component ratio remains as high as 0.724, while functional chain connectivity reliability has declined to near zero. Topological metrics overestimate actual functional status.	Relatively low. The state of each functional chain is discretely observable (operational/failed), and the network reliability can be traced precisely along its decline trajectory, with each phase transition linked to identifiable trigger events.

**Table 3:** Systematic comparison between existing metrics and functional chain connectivity reliability.

Metric	Key Assumptions	Scope	Dimensions	Heterogeneity	Dynamic Reconfig.	Data	Object	Granularity
K-terminal reliability [25]	Static topology, independent edge/node failure	Wired/static wireless network design	Single (prob.)	No	No	Adjacency matrix	Links/ connections	Network
Capacitated Resilience [10]	Fixed hierarchy, perfect nodes, split flows, no cascading	HetNet design optimization	Single [0, 1]	Partial	No	Topology + capacity + demand	End-to-end path	User→ Network
Mission Reliability [11]	Homogeneous units, fixed formation, independent failure	Homogeneous UAV formations	Single (prob.)	No	Partial	Unit reliability + formation	Formation structure	System
Network Resilience [12]	Spatio-temporal dynamics + stochastic geometry	Flying Ad Hoc Networks	Composite (perf.-time integral)	No	No	Topology snapshots + mission perf.	Network connectivity/completion rate	Network
SoS Resilience [13]	Task-capability- resource linear mapping	Unmanned SoS	Composite (perf. curve)	Yes	Yes	Task- capability- resource matrix	SoS capability	SoS
CSoS Robustness [4]	Directed heterogeneous graph, 4 node types, collaborative reconfig.	Combat SoS	Composite (ANOC)	Yes	Yes	Topology + capability attributes	Combat loops	Network
Functional Chain Conn. Reliability (This work)	OODA functional chains, 3 node types, 3 failure modes, Couzin-leader mobility, DRS	Heterogeneous MANET operation	Multi-metric	Yes	Yes	Topology + roles + mobility + failure + link reliability	Functional chain	Chain→ Network

**Table 4:** Notation and definition.

Notation	Definition
$G=\left(V,E\right)$	The Graph-theoretic model of the MANET with node set V and edge set E
$G_{fd}$	A fixed-formation heterogeneous MANET
$N_{i}$	The i-th node in G
$e_{ij}$	The directed link from source node $N_{i}$ to sink node $N_{j}$
$V_{type}$ & $E_{type}$	The predefined node and edge types
$N_{c}^{C}$	A decision node
$N_{s}^{S}$	A sensos node
$N_{w}^{W}$	An action execution node
$S_{N_{i}}$	The operational state of node $N_{i}$
$d_{ij}$	The Euclidean distance between node $N_{i}$ $\mathrm{and}\;N_{j}$
$l_{j}^{kc}$	The j-th functional chain in G
$l_{j,F}^{ck}$	The j-th functional chain is marked as failed
$Nei^{d_{Co}}$	The set of neighbor nodes that node $N_{i}$ needs to exclude
$Nei_{1}^{d_{At}}$	The set of neighbor nodes that belong to the same functional chain
$Nei_{2}^{d_{At}}$	The set of neighbor nodes that within the circular plane
$R_{e}\left(d_{ij}\left(t\right)\right)$	$\mathrm{The}\;\mathrm{reliability}\;\mathrm{of}\;\mathrm{link}\;e_{ij}$
$R_{e}^{Th}$	The link reliability threshold
$A_{RB}\left(t\right)$	The binary adjacency matrix
$V_{Cd}$	The set of candidate nodes
$Cl^{kc}\left(t\right)$	A cell array records functional-chain
$N_{kc}\left(t\right)$	The number of complete functional chains
$R_{kc}\left(t\right)$	The normalized connectivity reliability
NQ	The network quality
$R_{kc,K\%}\left(t\right)$	The K% connectivity reliability
$I_{kc}^{K\%}\left(t\right)$	An indicator function

**Table 5:** Simulation parameters and the corresponding values.

Parameter	Value	Parameter	Value
m	210	$\omega_{2}^{d_{At}}$	1
$m_{i}\left(i=1,2,3\right)$	70	$\omega_{i}$	1.5
$\max N_{kc}$	70	$\lambda_{i}$	$2.78\times 10^{-8}{\;s}^{-1}$
K	80	α	0.5
$d_{At}$	100 m	β	0.7
$d_{Co}$	20 m	$R_{e}^{Th}$	0.8
$d_{Th}$	70 m	$t_{mi}$	3600 s (1 h)
$v_{\min}$	5.56 m/s (20 km/h)	Δt	1 s
$v_{\max}$	16.67 m/s (60 km/h)	$N_{simu}$	100
$\omega_{1}^{d_{At}}$	3		

**Table 6:** Comparison of reliability metrics in simulation experiments.

Reliability Metrics	Control Group Network $G_{NDR}$	Dynamically Reconfigurable Network $G_{DR}$	Difference Value (Difference Ratio)
Number of functional chains $N_{kc}$	12.10 ± 0.673	32.77 ± 2.241	20.67 (170.83%)
Normalized connectivity reliability $R_{kc}$	0.1729 ± 0.010	0.4681 ± 0.032	0.2952 (170.73%)
Network quality NQ	1341.0494	2453.6241	1112.5747 (82.9630%)

## Data Availability

The data presented in this study are available on request from the corresponding author.

## Appendix A

Parametric Analysis Experimental Data.

**Table A1:** Effect of network scale on connectivity reliability metrics.

$N_{total}$	$R_{kc}$	$N_{kc}$	NQ
90	0.326 ± 0.044	9.78 ± 1.31	2220.2 ± 115.0
150	0.411 ± 0.037	20.56 ± 1.82	2366.9 ± 89.2
210	0.471 ± 0.029	32.95 ± 2.03	2461.2 ± 77.0
270	0.495 ± 0.031	44.55 ± 2.79	2523.7 ± 69.8
330	0.516 ± 0.024	56.78 ± 2.67	2587.9 ± 57.4

**Table A2:** Effect of maximum velocity on connectivity reliability metrics.

$v_{\max}$	$R_{kc}$	$N_{kc}$	NQ
11.11 m/s	0.585 ± 0.030	40.94 ± 2.10	3027.8 ± 58.5
13.89 m/s	0.519 ± 0.028	36.35 ± 1.98	2675.9 ± 67.9
16.67 m/s	0.471 ± 0.029	32.95 ± 2.03	2461.2 ± 77.0
19.44 m/s	0.405 ± 0.032	28.33 ± 2.27	2317.1 ± 81.6
22.22 m/s	0.296 ± 0.036	20.75 ± 2.50	2164.5 ± 79.9

**Table A3:** Effect of failure rate on connectivity reliability metrics.

$\lambda /\lambda_{0}$	$R_{kc}$	$N_{kc}$	NQ
0.1×	0.558 ± 0.028	39.09 ± 1.93	2581.2 ± 75.6
0.5×	0.536 ± 0.027	37.55 ± 1.90	2542.1 ± 72.0
1.0×	0.471 ± 0.029	32.95 ± 2.03	2461.2 ± 77.0
2.0×	0.381 ± 0.030	26.65 ± 2.11	2332.0 ± 79.9
5.0×	0.147 ± 0.024	10.30 ± 1.70	1986.6 ± 80.4

**Table A4:** Effect of communication range on connectivity reliability metrics.

$d_{Th}$	$R_{kc}$	$N_{kc}$	NQ
0.6× (42 m)	0.000 ± 0.000	0.00 ± 0.00	588.7 ± 32.2
0.8× (56 m)	0.302 ± 0.025	21.13 ± 1.71	1901.9 ± 82.8
1.0× (70 m)	0.471 ± 0.029	32.95 ± 2.03	2461.2 ± 77.0
1.2× (84 m)	0.606 ± 0.026	42.42 ± 1.80	2852.0 ± 54.7
1.5× (105 m)	0.725 ± 0.014	50.77 ± 0.97	3167.0 ± 29.8

**Table A5:** Effect of dynamic reconfiguration strategy on connectivity reliability metrics.

Strategy	$R_{kc}$	$N_{kc}$	NQ
S0: NoReconf	0.182 ± 0.008	12.73 ± 0.56	1357.7 ± 33.6
S1: Random	0.465 ± 0.032	32.52 ± 2.27	2458.2 ± 83.8
S2: Greedy	0.489 ± 0.028	34.23 ± 1.93	2491.5 ± 71.7
S3: MinMove	0.470 ± 0.032	32.88 ± 2.22	2465.2 ± 78.3
S4: DRS	0.473 ± 0.029	33.14 ± 2.00	2471.3 ± 75.4

**Table A6:** Effect of dynamic reconfiguration period on connectivity reliability metrics.

Period	$R_{kc}$	$N_{kc}$	NQ
1 s (real time)	0.478 ± 0.029	33.47 ± 2.02	2491.7 ± 73.2
5 s	0.499 ± 0.018	34.90 ± 1.29	2366.3 ± 61.0
10 s	0.503 ± 0.018	35.18 ± 1.28	2279.0 ± 56.0
15 s	0.528 ± 0.015	36.97 ± 1.07	2226.9 ± 53.3
20 s	0.510 ± 0.017	35.73 ± 1.19	2187.1 ± 51.3
